# An inner membrane protein is covalently attached to peptidoglycan in the γ-proteobacterium *Dickeya dadantii*

**DOI:** 10.1038/s42003-025-08488-9

**Published:** 2025-07-18

**Authors:** Xavier Nicolai, Yucheng Liang, Florence Ruaudel, Magdalena Narajczyk, Robert Czajkowski, Filippo Rusconi, Michel Arthur, Vladimir E. Shevchik

**Affiliations:** 1 CNRS UMR 5240 Microbiologie Adaptation et Pathogénie, Université Claude Bernard Lyon 1, INSA Lyon, Villeurbanne, France; 2 Centre de Recherche des Cordeliers, Sorbonne Université, INSERM, Université de Paris, Paris, France; 3 Bioimaging Laboratory, Faculty of Biology, University of Gdansk, Gdansk, Poland; 4 Laboratory of Biologically Active Compounds, Intercollegiate Faculty of Biotechnology UG and MUG, University of Gdansk, Gdansk, Poland; 5 GQE-Le Moulon/PA, INRAE, CNRS, AgroParisTech, IDEEV, Université Paris-Saclay, Gif-sur-Yvette, France

**Keywords:** Bacteria, Cellular microbiology

## Abstract

Gram-negative (diderm) bacteria possess a multilayered envelope comprising an inner membrane, a thin peptidoglycan (PG) layer and an outer membrane. In *Escherichia coli* and certain other γ-proteobacteria, including *Dickeya dadantii*, Braun lipoprotein, Lpp, covalently tethers the outer membrane to PG. Here, we show that in *D. dadantii* an inner membrane protein, OutB, is covalently attached to PG by the same catalytic mechanism as Lpp. Specifically, two L,D-transpeptidases, Ldt03 and Ldt84, catalyze protein attachment with a preference for muropeptide monomers and dimers, respectively. By altering the Lpp length, we show that the extended Lpp+21 enhances OutB attachment to PG, whereas the truncated LppΔ21 reduces it. Furthermore, we show that the PG-anchoring sequence of OutB tolerates substantial amino acid substitutions and allows PG-tethering of a periplasmic reporter protein, suggesting that other periplasmic and/or membrane proteins may also be tethered to PG in proteobacteria.

## Abstract (web summary)

In Gram-negative bacterium Dickeya dadantii, an inner membrane protein OutB is covalently attached to peptidoglycan by the same catalytic mechanism as Braun’s lipoprotein.

## Introduction

The envelope of Gram-negative (diderm) bacteria consists of an inner membrane (IM) and an outer membrane (OM) that delineate the periplasm, containing a thin layer of peptidoglycan (PG)^1^. PG is an essential component of the cell envelope, which mechanically sustains the turgor pressure of the cytoplasm^2^. The mesh-like structure of the PG macromolecule consists of glycan chains made of alternating *N*-acetylglucosamine (GlcNAc) and *N*-acetylmuramic acid (MurNAc) residues linked by β-1,4-bonds^3^. The lactoyl group of MurNAc residues is linked via an amide bond to a pentapeptide stem, which in *Escherichia coli* consists of the sequence L-Ala^1^-D-Glu^2^-DAP^3^-D-Ala^4^-D-Ala^5^, wherein DAP is diaminopimelic acid^4^. In this bacterium, the stem peptides are mainly cross-linked to each other via D-Ala^4^ → DAP^3^ amide bonds (4 → 3 cross-links) formed by D,D-transpeptidases of the penicillin-binding protein (PBP) family^5,6^. In addition, L,D-transpeptidases form DAP^3^ → DAP^3^ (3 → 3) cross-links, which account for 3–10% of the cross-links, depending on the growth phase^7,8^. Although not essential under standard laboratory conditions, the 3 → 3 cross-links play an important role in β-lactam resistance and PG homeostasis^9,10^. In certain α- and β-proteobacteria, an alternative type of L,D-transpeptidase catalyzes the formation of 1 → 3-type cross-links between L-Ala^1^ and DAP^3^^11,12^. Despite the different linking sites, 3 → 3 *versus* 1 → 3, both these Ldt families share a YkuD-like catalytic domain (PF03734). In addition, another class of 3 → 3 cross-linking Ldts possess a structurally unrelated VanW-type catalytic domain (PF04294)^13^.

The attachment of the PG layer to both the IM and OM is essential to maintain the integrity of the cell envelope of diderm bacteria. In the majority of Gracilicutes, two OM proteins, OmpA and Pal, non-covalently attach the OM to the PG via their conserved PG-binding domains^14–17^. The Tol-Pal trans-envelope complex links the PG to both the IM and OM via the Pal and TolR proteins, respectively^18–20^. Furthermore, in a subclade of γ-proteobacteria, including *E. coli*, the Braun lipoprotein (Lpp) covalently tethers the OM to the PG layer^14,21^. The N-terminal Cys residue of mature Lpp is acylated by three fatty acids, which are embedded in the inner leaflet of the OM. The side-chain ε-amino group of the C-terminal Lys residue of Lpp (Lys^58^) is covalently linked to the α-carbonyl of the DAP residue of PG tripeptide stems^22,23^. This cross-linking reaction is catalyzed by specialized L,D-transpeptidases, ErfK (LdtA), YbiS (LdtB), or YcfS (LdtC)^24^. Three Lpp molecules form together a trimeric helix that tethers the OM to the PG layer^25^. Lpp is the most abundant protein in *E. coli* (10^6^ copies per cell), about one-third of which is attached to PG^22,26^. For the past five decades, Lpp has remained the sole protein known to be covalently linked to PG in diderm bacteria. However, two recent studies have shown that in some α- and γ-proteobacteria lacking any *lpp* ortholog, certain β-barrel outer membrane proteins (OMPs) are covalently linked to the DAP residue of the stem peptide via their N-terminal Ala or Gly residue^27,28^. These OMPs provide an alternative mechanism for maintaining envelope stability through the tethering of the OM to the PG. Moreover, in the γ-proteobacterium *Coxiella burnetii*, the OM lipoprotein LimB, a functional analog of Lpp, is attached to PG through an internal lysine residue^27^. In addition to its osmoprotective role, the PG layer serves as a scaffold for the non-covalent attachment of various proteins and protein complexes. For instance, many trans-envelope machineries that span the cell envelope of diderm bacteria are non-covalently linked to the cell wall by specialized PG-binding domains of various types, such as AMIN, LysM, SPOR, or OmpA-like^29–32^.

**Fig. 1:**
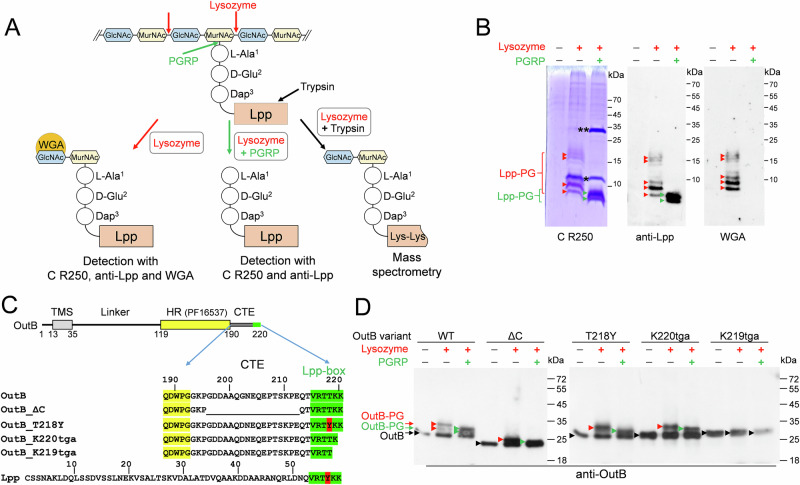
Covalent attachment of Lpp_Dda_ and OutB to PG in *D. dadantii.* **A** Schematic of the analysis of PG purified from *D. dadantii*. The cleavage sites of lysozyme, PGRP, and trypsin are shown with red, green, and black arrows, respectively. Abbreviations: GlcNAc *N*-acetylglucosamine, MurNAc *N*-acetylmuramic acid, DAP diaminopimelic acid. Wheat germ agglutinin (WGA) recognizes GlcNAc residues. **B** SDS-PAGE and Western blot analyses of the protein content of *D. dadantii* PG. PG was digested or not with lysozyme and PGRP amidase as indicated. Lpp-PG adducts attached to muropeptides were detected with Coomassie R250 (C R250) (left panel), anti-Lpp antibodies (middle panel), or WGA (right panel). Lpp-PG adducts generated by lysozyme and PGRP are shown with red and green arrowheads, respectively. The positions of lysozyme and PGRP are indicated by one and two asterisks, respectively. **C** Domain organization of OutB. OutB comprises a transmembrane segment (TMS), a periplasmic linker, a homology region (HR) (in yellow), and a C-terminal extension (CTE) carrying the Lpp box (in green). OutB variants carrying mutations in the CTE or Lpp-box are aligned together with the mature Lpp of *D. dadantii*. Sequence similarity between OutB and Lpp_Dd_ is limited to the Lpp box. **D** Western blots of PG purified from *D. dadantii* expressing the indicated OutB variants (shown in **C**). PG was digested or not with lysozyme and PGRP amidase and probed with anti-OutB antibodies. OutB-PG adducts generated by lysozyme and PGRP are indicated with red and green arrowheads, respectively. The positions of “free” OutB forms are shown with black arrowheads.

*Dickeya dadantii* is a plant pathogenic γ-proteobacterium that secretes an array of virulence effectors via a type 2 secretion system (T2SS), termed Out^33^. The Out system is a trans-envelope complex comprising fourteen proteins: OutB to OutM, OutO, and OutS^34^. Among these proteins, OutB acts as a scaffolding protein facilitating the assembly of the OM pore formed by secretin OutD^35^. OutB is anchored to the IM by its N-terminal transmembrane segment (TMS), followed by a 75-residue linker and the Homology Region (HR) (PF16537), ended by a 30-residue C-terminal extension (CTE) (Fig. 1C).

Here, we employed a combination of biochemical, genetic, and mass spectrometry (MS) analyses to demonstrate that OutB possesses a PG-anchoring consensus sequence (Lpp-like box) and that its C-terminal Lys residue is covalently attached to the stem peptide of the *D. dadantii* PG by the same catalytic mechanism as Lpp. Two L,D-transpeptidases, Ldt03 and Ldt84, were identified as being involved in this process, with a preference for attaching the protein to muropeptide monomers and 4 → 3 cross-linked dimers, respectively. To determine how spatial constraints across the periplasm control OutB attachment to PG, we generated Lpp variants of different lengths that displace the PG layer in the periplasm and found that the extended Lpp + 21 enhances OutB attachment to PG, whereas the shortened LppΔ21 has the opposite effect, reducing it. Mutagenesis analysis of the Lpp-box showed that it tolerates substantial amino acid substitutions and allows the attachment of a periplasmic reporter protein to the PG. These data suggest that other periplasmic and/or membrane proteins exposed to the periplasm with a C-terminal Lys residue may be covalently attached to the PG in *D. dadantii* and possibly in other proteobacteria.

## Results

### Lpp_Dd_ is covalently linked to the *D. dadantii* PG

Five of the six C-terminal residues of OutB (VRTTKK) are identical to those of *D. dadantii* Lpp, Lpp_Dd_ (VRTYKK). This prompted us to examine whether OutB is covalently attached to the PG layer, as would be the case for Lpp_Dd_. To this end, the PG of *D. dadantii* was purified by the hot SDS procedure, which eliminates all the proteins except those covalently linked to the PG. The PG was then digested with lysozyme, that cleaves the MurNAc-GlcNAc β-1,4 bonds (Fig. 1A), thereby releasing disaccharide-peptide fragments, including those covalently linked to Lpp or, potentially, to OutB. Accordingly, SDS-PAGE and immunoblotting of this PG sample revealed a series of protein bands reactive with anti-Lpp antibodies and with the wheat germ agglutinin (WGA) (Fig. 1A, B). WGA recognizes GlcNAc residues^36^, thereby indicating the presence of Lpp covalently linked to muropeptides. Consequently, digestion of the PG with PGRP, an amidase from the weevil *Sitophilus zeamais* that cleaves the MurNAc-L-Ala^1^ amide bond (Fig. 1A)^37^, resulted in the loss of the WGA-reactive species and the formation of two higher-mobility Lpp adducts, involving only stem peptide moieties (Fig. 1B). These results suggest that Lpp_Dd_ is covalently linked to two distinct types of stem peptides.

To characterize the molecular link connecting Lpp_Dd_ and OutB to PG, the structure of *D. dadantii* PG was analyzed by MS and found to be similar to that of *E. coli* (Fig. S1 and Table S1). Briefly, the main monomers consisted of the disaccharide GlcNAc-MurNAc and a tripeptide (L-Ala^1^-D-Glu^2^-DAP^3^) or a tetrapeptide (L-Ala^1^-D-Glu^2^-DAP^3^-D-Ala^4^) stem. The main dimers contained a tetrapeptide stem linked to a tripeptide (Tetra→Tri dimer) or to a tetrapeptide (Tetra→Tetra dimer) stem by a D-Ala^4^→Dap^3^ 4 → 3 cross-link formed by the D,D-transpeptidase activity of PBPs (Table S1). A disaccharide-tripeptide monomer and 4 → 3 cross-linked dimer substituted by a Lys-Lys dipeptide were detected (Fig. S2). These muropeptides correspond to the products of protein digestion by trypsin, leaving the two C-terminal Lys residues bound to a peptide stem, indicative of partial cleavage of the Lys-Lys peptide bond by trypsin. Additionally, minor quantities of muropeptides comprising a single Lys residue were detected. Since Lpp_Dd_ and OutB both share the identical Lys-Lys C-terminal sequence (Fig. 1C), the Tri→Lys-Lys and Tetra→Tri→Lys-Lys molecular species may potentially originate from the digestion of both PG-bound Lpp_Dd_ and OutB adducts by trypsin. To investigate this possibility, we conducted a PG analysis of the *D. dadantii lpp* mutant. The Tri→Lys-Lys and Tetra→Tri→Lys-Lys were not detected in this PG indicating that the sensitivity of the MS analysis is sufficient only to detect muropeptide-linked adducts derived from the highly abundant Lpp_Dd_ protein.

### OutB is covalently linked to the *D. dadantii* PG

The PG preparations used above to identify Lpp-muropeptide adducts were subsequently probed by immunoblotting for the presence of OutB covalently linked to PG (Fig. 1D, left panel). Notably, a certain quantity of OutB was detected in the intact, undigested PG (Fig. 1D, lane without addition of lysozyme or PGRP). This was tentatively attributed to the “free” unbound form of OutB that remained entrapped in the intact sacculi during PG extraction, but escaped from them during SDS-PAGE. Such a “free” form was not observed with Lpp (Fig. 1B), which has a much smaller size (58 *versus* 220 residues) and, in contrast to OutB, is naturally located outside the sacculi. Digestion of the PG with lysozyme produced two additional slower-migrating OutB species that could correspond to OutB linked to muropeptides (Fig. 1D, left panel). Accordingly, additional digestion with PGRP, which removes saccharide moieties, resulted in OutB adducts with slightly increased mobility (Fig. 1D, left panel). These species could correspond to OutB linked to peptide moieties of PG.

To further test this hypothesis, we attempted to probe these OutB adducts with WGA. However, its detection was hampered by high background noise generated by very abundant Lpp-linked muropeptides. To overcome this issue, PG extracted from *D. dadantii lpp* mutant ectopically expressing *outB* was analyzed. WGA-reactive OutB species was detected in this PG digested with lysozyme (Fig. S3), indicating the presence of GlcNAc-containing muropeptides linked to OutB. Collectively, these results show that OutB is covalently linked to the peptide moieties of PG.

### The Lpp box of OutB is necessary and sufficient for the covalent attachment to PG

The 6-residue Lpp-like box of OutB is located at the C-terminal end of the CTE and is preceded by a 22-residue region following the HR domain (Fig. 1C). This part of the CTE is rich in charged residues and shares some features with sugar-binding motifs^38^. However, removal of a substantial portion of the CTE outside the Lpp-box (residues G^196^ to E^212^) did not prevent covalent attachment of the resulting OutBΔC to PG (Fig. 1C, D), indicating that the deleted region is not essential for this purpose.

**Fig. 2:**
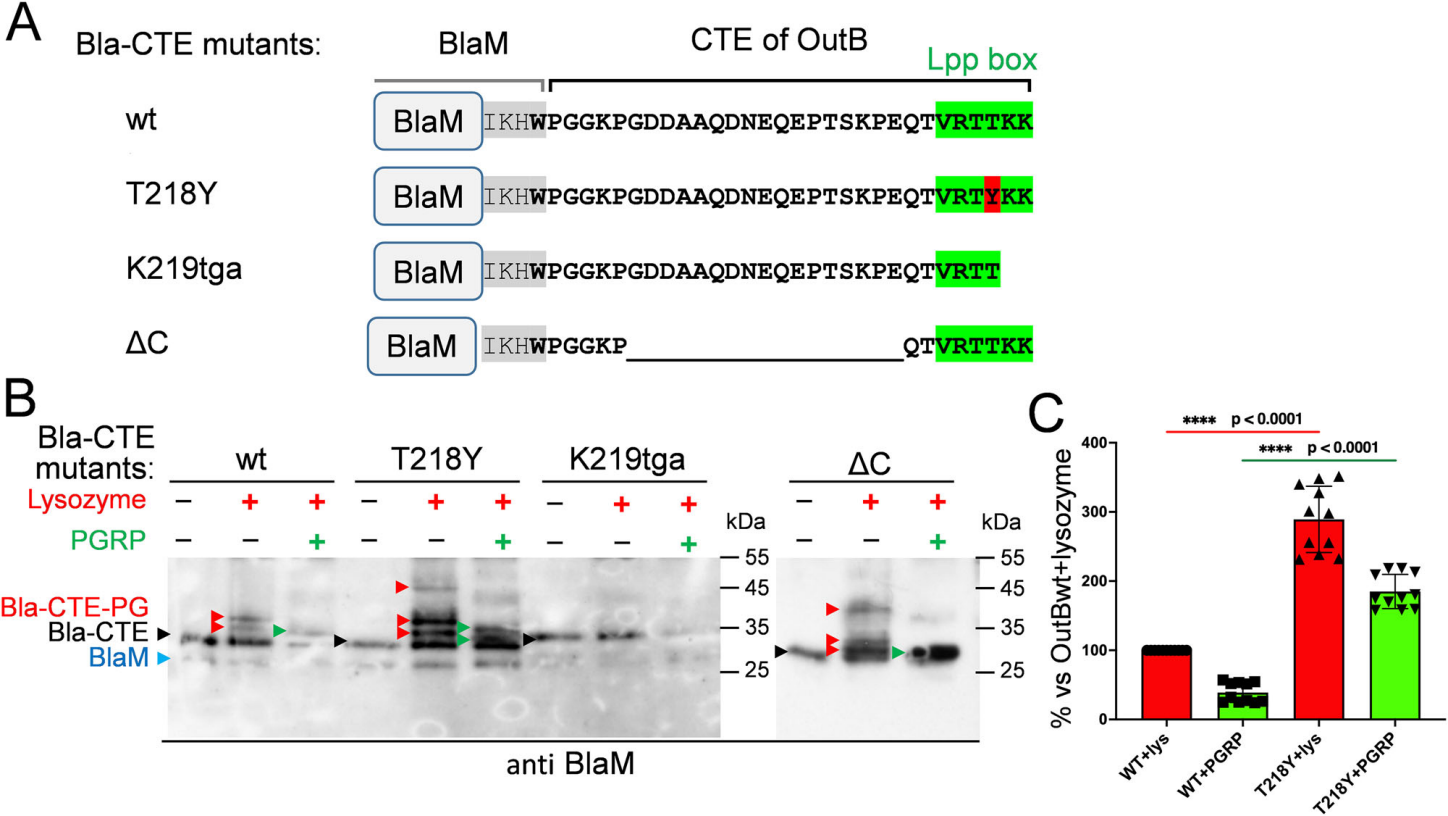
Lpp-box of OutB allows attachment of the β-lactamase BlaM to PG. **A** Schematic of the BlaM-CTE fusions. The C-terminus of the native BlaM is highlighted in gray; the rest of the sequence corresponds to the CTE of OutB (residues P191 to K220). The Lpp-like box is highlighted in green and the T218Y substitution is in red. The numbering of residues substituted in BlaM-CTE mutants is that of the OutB CTE. **B** Western blot analysis of PG purified from *D. dadantii* expressing the indicated Bla-CTE variants. PG was digested or not with lysozyme and PGRP amidase and probed with anti-BlaM antibodies. The positions of BlaM and “free” forms of Bla-CTE fusions are shown with blue and black arrowheads, respectively. Bla-CTE-PG adducts generated by lysozyme and PGRP are indicated with red and green arrowheads, respectively. **C** Relative amounts of muropeptide-linked adducts of Bla-CTE_wt and Bla-CTE_T218Y generated by lysozyme (in red) and PGRP (in green). They correspond to the species shown respectively with red and green arrows in (**B**). The data from two independent biological experiments were analyzed with PRISM software using two-sample Mann–Whitney test by comparing the values of muropeptide-linked adducts of Bla-CTE_T218Y to those of Bla-CTE_wt considered as 100% (red bar). Median and quartiles are shown. **** denote statistically significant differences with *P* values < 0.0001. Source data are provided in Supplementary Data 1.

To further address the issue, either the full-length 30-residue CTE of OutB or only its truncated 13-residue portion (as in OutBΔC) was fused to the C-terminus of β-lactamase BlaM, a bona fide periplasmic protein, thereby forming Bla-CTE and Bla-CTEΔC, respectively (Fig. 2A). Immunoblotting showed that both hybrids are covalently linked to PG (Fig. 2B). Indeed, digestion of the corresponding PG with lysozyme yielded three BlaM-reactive adducts of lower electrophoretic mobility, consistent with the presence of covalently attached muropeptides. Addition of PGRP amidase generated a BlaM species of a smaller apparent size, indicating elimination of glycan moieties (Fig. 2B). These data show that the Lpp-box of OutB constitutes a genuine PG-linking motif that allows covalent attachment of the periplasmic reporter protein to the *D. dadantii* PG.

### The Lpp box can tolerate substantial amino acid substitutions

Unlike *E. coli* Lpp, which carries a C-terminal Arg-Lys pair, the C-termini of Lpp_Dd_ and OutB contain two Lys residues (Fig. 1C). To evaluate the role of these residues in the attachment of OutB to PG, an Opal stop codon was introduced in place of the codons encoding Lys^219^ or Lys^220^. As expected, deletion of the two C-terminal Lys abolished the attachment of the resulting OutB_K219tga to PG (Fig. 1D). However, OutB_K220tga, which lacks the C-terminal Lys^220^, was tethered to PG at the wild-type level (Fig. 1D). This suggests that the remaining penultimate Lys^219^, which has become the C-terminal residue, may link OutB to PG. Strikingly, the equivalent substitution in Lpp (loss of the C-terminal Lys^58^) showed about 50-fold reduction in the amount of PG-linked LppΔ58K (Fig. S4A, B). Accordingly, LppΔ58K did not improve the resistance of the *D. dadantii lpp* mutant to SDS (Fig. S4C). These results show that a single Lys residue at the C-terminus of the Lpp box is both necessary and sufficient for covalent attachment to PG, but its reactivity varies depending on the protein.

The sole difference between the Lpp-box of OutB and Lpp_Dd_ is the presence of Thr *versus* Tyr at the antepenultimate position (Fig. 1C). The above data indicated that the PG-linking activity of LppΔ58K is more drastically affected than that of OutB_K220tga. To render the truncated Lpp-box of LppΔ58K identical to that of OutB_K220tga (VRTYK *versus* VRTTK), a Y56T substitution was introduced into LppΔ58K. This did not improve the attachment of the resulting LppY56T/Δ58K to PG or its activity in the SDS susceptibility assay with the *D. dadantii lpp* mutant (Fig. S4). More markedly, the Y56T substitution in the full-length Lpp notably reduced the attachment of the resulting LppY56T to PG and diminished its efficacy in the SDS susceptibility assay (Fig. S4). In contrast, the inverse T218Y substitution in the Bla-CTE enhanced the attachment of the protein to PG (Fig. 2B, C). Thus, the polymorphism of the antepenultimate position, Thr (OutB) *versus* Tyr (Lpp_Dd_), has some effect on the tethering of these proteins to PG. Taken together, these results show that the Lpp-box can tolerate substantial amino acid substitutions, depending on the tethered protein.

### Anchoring of OutB to the inner membrane is not essential for its linkage to PG

The N-terminal IM anchor is critical for the scaffolding function of OutB towards the secretin OutD^35^. To assess the role of this TMS in the covalent attachment of OutB to PG, a soluble periplasmic variant, OutB_SP, was generated, in which the TMS was replaced by a cleavable signal peptide (Fig. S5A). In contrast to the full-length OutB and other OutB variants, no “free” form of OutB_SP was detected in the intact, undigested PG (Fig. S5B), indicating that OutB_SP is too small (~19 kDa) to be retained by the PG meshwork. Lysozyme digestion of this PG sample yielded an anti-OutB reactive adduct of ~30 kDa, whereas the addition of PGRP amidase resulted in a notable reduction in its amount and the appearance of a 25 kDa species (Fig. S5B). These data show that the anchoring of OutB to the IM is not mandatory for the tethering to the PG.

### Alteration of the Lpp_Dd_ length affects the integrity of the *D. dadantii* envelope

**Fig. 3:**
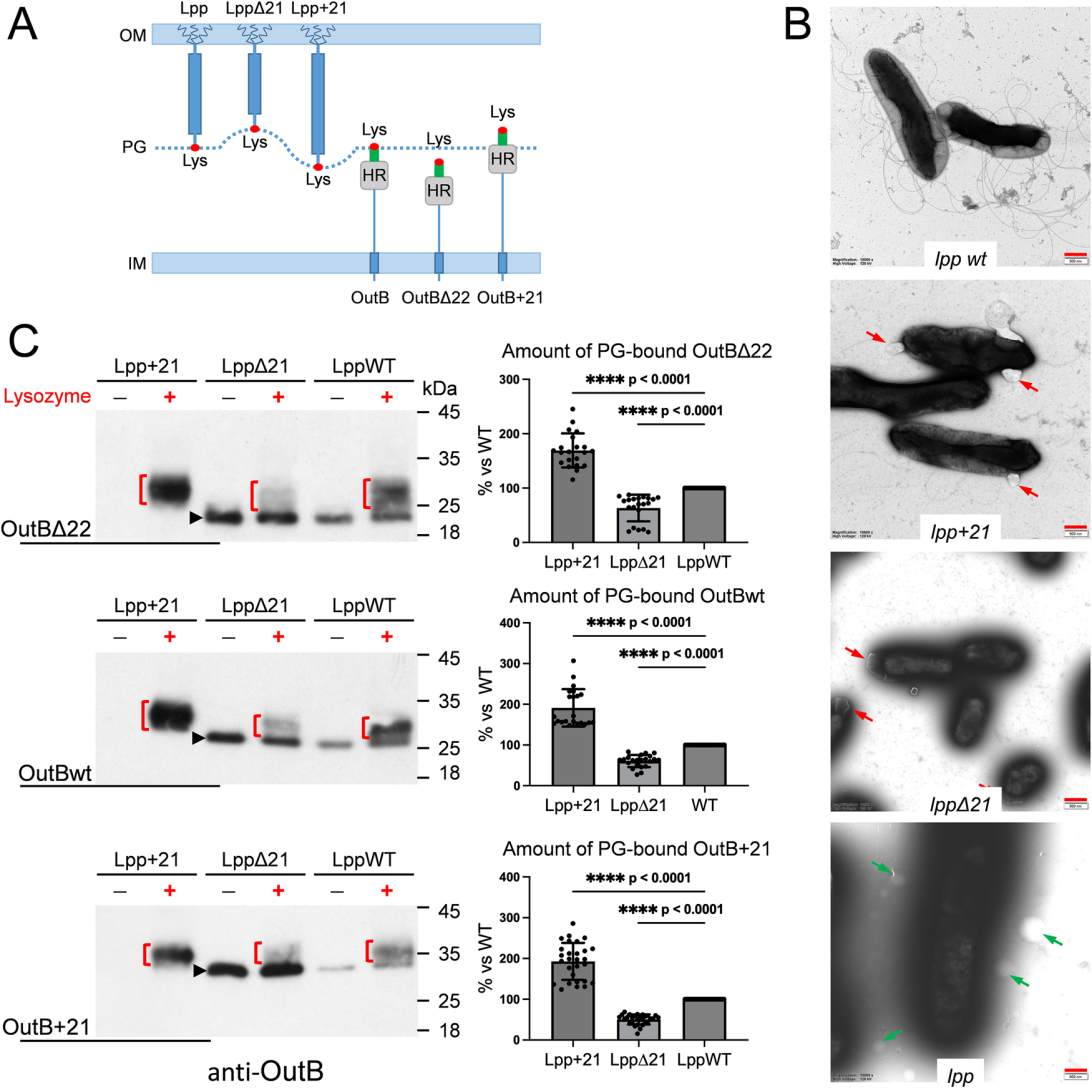
Altering the Lpp_Dd_ length affects OutB attachment to PG. **A** Schematic of the Lpp and OutB length variants used in the study (see Figs. S6A and S9A for details). **B** Negative stain TEM of the indicated *D. dadantii lpp* mutant strains. Red bars (lower right corner) show 500 nm scale; magnification is ×18,500. Blebs formed by *lpp*Δ*21* and *lpp* + *21* cells are shown with red arrows and vesicles around *lpp* mutant, with green arrows. **C** OutBΔ22, OutBwt, and OutB+21 length variants (indicated on the left) were expressed in *D. dadantii lpp* length mutants (*lppWT*, *lppΔ21*, and *lpp* + *21*, indicated on the top of Western blots). PG was extracted from these nine strains, digested or not with lysozyme and probed with anti-OutB. OutB-PG adducts generated by lysozyme are shown with red brackets. “Free” forms of OutB variants are indicated with black arrowheads. The data from two independent biological experiments were analyzed with PRISM software using two-sample Mann–Whitney test by comparing the values in *lpp* + *21* and *lpp*Δ*21* mutants to those in the wild-type strain considered as 100%. Histograms show relative amounts of muropeptide-linked adducts for each OutB length variant in each *D. dadantii lpp* length mutant (shown with red brackets in the corresponding Western blots). Median and quartiles are shown. **** denote statistically significant differences with *P* values < 0.0001. Source data are provided in Supplementary Data 1.

In *E. coli* and some other γ-proteobacteria, Lpp tethers the PG layer to the OM, thereby controlling the size of the periplasm. Altering the length of Lpp has been shown to alter the width of the periplasm and the distance between the PG layer and each of the two cell membranes^39–41^. We wondered whether such variations in the Lpp_Dd_ length would affect the attachment of OutB to PG. At the same time, we tested whether alterations in the length of OutB could also affect its linkage to PG (Fig. 3A).

In this aim, we constructed *D. dadantii* mutants producing a shortened or a lengthened Lpp_Dd_ variant, LppΔ21 and Lpp+21, respectively. The *lpp*Δ*21* and *lpp* + *21* alleles were introduced into the *D. dadantii* chromosome in place of the wild-type *lpp* gene (Fig. S6A, B). Analysis of PG from these strains revealed that the apparent size of the LppΔ21 and Lpp + 21 species linked to muropeptides varied according to the length of each variant (Fig. S6C). *D. dadantii lppΔ21* formed very mucoid colonies and was non-motile, but had wild-type resistance to SDS (Fig. S6D, E). In contrast, *D. dadantii lpp* + *21* showed increased susceptibility to SDS, yet its motility was barely affected (Fig. S6D, E). *D. dadantii lppΔ21* cells were significantly shorter compared to the wild-type strain and the *lpp* + *21* mutant (Figs. 3B and S7). Both the *lpp*Δ*21* and *lpp* + *21* mutants produced a sort of blebs, which remained attached to the cells, unlike the vesicles in the *lpp* mutant (Fig. 3B). In scanning EM, such envelope defects were visible as cavities at the cell poles of the *lpp*Δ*21* mutant (Fig. S8). Some of these phenotypes have been previously observed in *E. coli* and *Salmonella enterica* mutants expressing Lpp-length variants^39–42^.

### Increasing Lpp length improves OutB attachment to PG

Subsequently, to evaluate the impact of OutB and Lpp length alterations on OutB tethering to PG, we constructed OutB variants with altered length, OutBΔ22 and OutB+21 (Figs. 3A and S9A) and combined them with the Lpp-length variants. In this way, the *outB*, *outBΔ22*, and *outB* + *21* genes were ectopically expressed in the *D. dadantii* wild-type, *lpp*Δ*21*, and *lpp* + *21* strains. The PG from these strains (nine combinations) was digested with lysozyme and analyzed by immunoblotting with anti-OutB (Figs. 3C and S9B). This analysis revealed that the quantity of OutB-PG species steadily increased as a function of Lpp length, from LppΔ21 to Lpp and to Lpp + 21, irrespective of the length of OutB itself (OutBΔ22, OutBwt, or OutB + 21) (Figs. 3C and S9B, C). Specifically, the highest amount of muropeptide-bound OutB variants was observed in *D. dadantii lpp* + *21*, while their lowest amount was detected in *D. dadantii lppΔ21*. These results suggest that the length of Lpp, which controls the width of the periplasm, is a critical factor influencing the accessibility of the Lpp-box of OutB to PG.

It is noteworthy that the “free” forms of OutB-length variants showed an inverse trend. Their levels gradually decreased as a function of Lpp length in the series of strains, from *lpp*Δ*21* to *lpp* and to *lpp* + *21* (Fig. 3C), consistent with a more massive linking of OutB in the *lpp* + *21* mutant. It is also possible that in the presence of LppΔ21 and Lpp+21, the PG meshwork is, respectively, more and less densely fitted than with the wild-type Lpp.

### Lpp_Dd_ and OutB are covalently tethered to PG by two L,D-transpeptidases with partially redundant functions

**Fig. 4:**
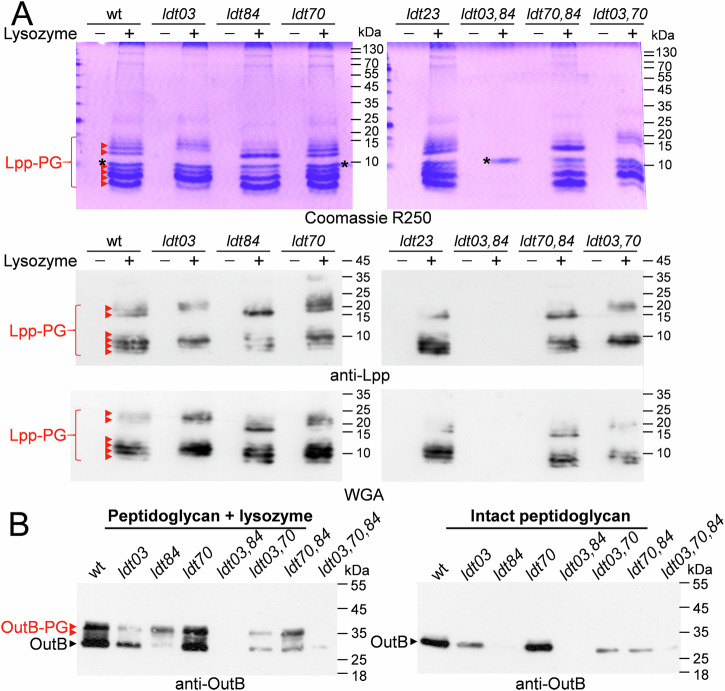
L,D-transpeptidases Ldt03 and Ldt84 are required for covalent attachment of Lpp and OutB to PG in *D. dadantii.* **A** SDS-PAGE and Western blot analyses of PG purified from the wild type (wt) *D. dadantii* and the indicated *ldt* mutants. PG was digested or not with lysozyme and Lpp-PG species were detected with Coomassie R250 (upper panel), anti-Lpp antibodies (middle panel), or with WGA (lower panel); they are shown with red arrowheads. The position of lysozyme is indicated with an asterisk. Some other double and triple *ldt* mutants are shown on Fig. S11. **B** The same PG samples as in panel A were probed with anti-OutB antibodies. “Free” form of OutB is shown with black arrowheads and OutB-PG adducts generated by lysozyme are indicated with red arrowheads.

Analysis of the *D. dadantii* 3937 genome (asap.genetics.wisc.edu) revealed the presence of four genes encoding putative L,D-transpeptidases, which carry a characteristic YkuD domain, ABF-0016403, ABF-0020084, ABF-0020070, and ABF-0046523, hereafter referred to as *ldt03, ldt84, ldt70,* and *ldt23*, respectively (Fig. S10). To identify the Ldts that are able to link Lpp_Dd_ and OutB to the PG, we constructed *D. dadantii* mutants with single and double deletions of these genes. SDS-PAGE and Western blot analyses of PG from these strains showed that the deletion of both *ldt03* and *ldt84* is necessary to suppress the attachment of Lpp_Dd_ and OutB to PG (Figs. 4 and S11A). Accordingly, the loss of Lpp_Dd_ tethering to PG upon the deletion of both *ldt03* and *ldt84* resulted in an increased SDS susceptibility of the double mutant (Fig. S11B). Markedly, the residual SDS resistance of *D. dadantii ldt03 ldt84* was notably higher than that of the *lpp* mutant, suggesting that the fraction of Lpp that is not covalently linked to PG plays a role in maintaining the integrity of the cell envelope.

Deletion of only *ldt03* or *ldt84* differently affected the abundance of various Lpp-PG and OutB-PG species (Figs. 4 and S11A), indicating that these enzymes exhibit certain degree of specificity for the PG-linking site. Consistent with this hypothesis, ectopic expression of *ldt03* in the double *ldt03 ldt84* mutant resulted in an Lpp-PG pattern similar to that of the *ldt84* mutant, and conversely, expression of *ldt84* yielded an Lpp-PG pattern similar to that of the *ldt03* minus strain (Fig. S12A). Likewise, ectopic expression of either *ldt03* or *ldt84* in the wild-type *D. dadantii* was found to increase the amount of PG-bound OutB, yet more obviously with *ldt03* (Fig. S12B).

Deletion of the *ldt70* or *ldt23* genes, either alone or in combination with *ldt03* or *ldt84*, did not appear to affect the Lpp-PG and OutB-PG patterns or the SDS susceptibility of the corresponding mutants (Figs. 4A and S11). The catalytic site of Ldt23 is similar to that of LdtF/DapA (YafK) that catalyzes the cleavage of Lpp from the stem peptide in *E. coli* (Fig. S10)^43,44^. Ldt70 shows the same domain organization as LdtD (YcbB), which is responsible for the 3 → 3 cross-links of stem peptides (Fig. S10)^45^. It seems plausible that Ldt23 and Ldt70 perform analogous functions to their respective *E. coli* orthologs.

### Ldt03 and Ldt84 display specificity for the nature of stem peptide

Given the notable dissimilarity between the Lpp-PG patterns produced by Ldt03 and Ldt84, we sought to elucidate the underlying molecular basis of these Ldt-specific patterns. In this order, MS analysis of PG from *D. dadantii ldt03* and *ldt84* mutant strains was performed. Muropeptides derived from PG were subjected to *rp*HPLC separation, and molecular species bearing the Lys-Lys moiety of Lpp were identified by MS (Fig. S13A). The relative abundance of each species was estimated based on their respective ion current intensities (Fig. S13B). This analysis revealed that the monomeric (Tri→Lys-Lys) muropeptide is predominant in the *D. dadantii ldt84* mutant (Ldt03+), (79.1% ± 6.3%), whereas the dimeric (Tetra→Tri→Lys-Lys) muropeptide is predominant in the *D. dadantii ldt03* mutant (Ldt84+) (72.0% ± 9.8%) (Fig. S13B). In agreement with this result, SDS-PAGE analysis of PGRP-digested PG from the *D. dadantii lpp ldt03 ldt84* triple mutant, which ectopically expresses *lpp-strep* with either *ldt03* or *ldt84*, resolved two Lpp-PG species consistent with those attached to monomeric and dimeric PG stem peptides (Fig. S13C). Once again, the putative monomer- and dimer-linked Lpp species were more abundant with Ldt03 and Ldt84, respectively. Taken together, these results show that Ldt03 and Ldt84 have a preference for tethering Lpp to monomeric and 4 → 3 cross-linked peptide stems, respectively.

To assess the extent of this phenomenon, three *E. coli* L,D-transpeptidases that attach Lpp to PG, ErfK (LdtA), YbiS (LdtB), and YcfS (LdtC)^24^, were expressed in the *D. dadantii ldt03 ldt84* mutant. Markedly, ErfK (LdtA) produced the Lpp-muropeptide pattern similar to that of Ldt84, with the predominance of the dimeric peptide, whereas the Lpp-muropeptide pattern of YbiS (LdtB) was similar to that of Ldt03 (Fig. S12A). These data suggest that the *E. coli* Ldts also show a preference for the type of peptide stem.

### OutB is expressed in response to envelope stress associated with the plant infection process

OutB has been shown to act as a scaffolding protein that facilitates the assembly of the secretin OutD pore^35^. The expression of *outD* is co-regulated with that of other genes of the *outC-outM* operon encoding the T2SS machinery, whereas *outB* constitutes independent transcription unit and its regulation remains unclear^46–48^. To gain further insight, we examined the production of OutB in a series of *D. dadantii* regulatory mutants. Strikingly, high levels of OutB were observed in the *phoP* and *ompR* mutants, but not in the *kdgR* or *pecS* mutants, as was the case with OutD (Fig. S14). The EnvZ/OmpR and PhoP/PhoQ systems are sensors of cell envelope and osmotic stresses^49^. Consistent with this, OutB was massively produced at high osmolarity (0.2 M NaCl) (Fig. S14A). These results suggest that OutB may play a particular role in response to envelope stress. *D. dadantii* T2SS secretes a number of pectinases causing plant tissue maceration^33^. Consistent with this, high levels of both OutB and OutD were detected in *D. dadantii* cells collected from rotted chicory leaves (Fig. S14B). These data show that during plant infection, OutB functions in concert with the cognate secretin OutD, which forms large multimeric pores in the OM to facilitate secretion of virulence factors by the T2SS.

## Discussion

In this study, we showed that the inner membrane protein OutB, a component of the *D. dadantii* T2SS, is covalently attached to PG by the same mechanism as Lpp. The site of attachment of OutB to the stem peptide of PG is located at the C-terminus of OutB and is nearly identical to that of Lpp. The originality of this finding lies in the substantial differences between OutB and a few other proteins known to be covalently linked to PG in diderm bacteria. Indeed, Lpp and β-barrel OMPs are highly abundant proteins that tether the OM to the PG layer^23,26–28^. In contrast, OutB is an IM protein that binds the PG layer from an opposite side of the periplasm. Moreover, under standard laboratory conditions, OutB is moderately expressed, yet its production increases at high osmolarity and in the infected plant tissues. Interestingly, an increased level of OutB in the *phoP* and *ompR* regulatory mutants (envelope stress markers) appears to be uncoupled from that of the cognate secretin OutD (Fig. S14B). This suggests that OutB may play an additional role and contribute to envelope integrity during the osmotic adaptation of *Dickeya* to its various environmental niches, such as soil, lakes, insects, and plants^50,51^.

We showed that two L,D-transpeptidases of *D. dadantii*, Ldt03 and Ldt84, catalyze the attachment of OutB and Lpp to PG. The majority of reported Ldts possess a YkuD catalytic domain (PF03734). The Ldts of the VanW family (PF04294) are much less prevalent and largely restricted to Gram-positive bacteria (monoderms)^13^. No Ldt of the VanW family was identified in *D. dadantii*. Proteobacteria possess a variable number of YkuD-type L,D-transpeptidases, ranging from only one in *Neisseria gonorrhoeae* and *Helicobacter pylori* to 10 and 14 in *Legionella pneumophila* and *Agrobacterium tumefaciens*, respectively^27,52^. The *E. coli* genome encodes six proteins with a YkuD-type catalytic domain. Three of these enzymes, ErfK (LdtA), YbiS (LdtB), and YcfS (LdtC), are responsible for the tethering of Lpp to PG^24^. The presence of multiple Ldts involved in the attachment of Lpp to PG may be attributed to their distinctive enzymatic properties and/or regulatory mechanisms^24^. *D. dadantii* possesses four YkuD-type enzymes, Ldt03, Ldt84, Ldt70, and Ldt23. We provide evidence that two of them, Ldt03 and Ldt84, attach Lpp and OutB to PG and show a clear preference for specific types of muropeptides, monomeric and dimeric peptide stems, respectively. Moreover, we showed that the *E. coli* L,D-transpeptidases that attach Lpp to PG also exhibit a preference for the type of peptide stem (Fig. S12A). To the best of our knowledge, this study is the first demonstration of Ldt specificity for a particular type of peptide stem.

In comparison with a few other known proteins covalently linked to the PG of diderm bacteria, OutB adopts a rather unusual topology. In OutB, the Lpp-box is grafted onto the periplasmic HR domain, which in turn is connected to the IM anchor by a 75-residue linker. The length of this linker varies even among closely related *Dickeya* species. For example, it is 83 residues in *D. dadantii subsp. dieffenbachiae* and 61 residues in *D. solani*. Consistent with this, we showed that a moderate truncation or extension of this linker (22 and 21 residues, respectively) has no discernible impact on OutB attachment to PG. In contrast, the construction of *D. dadantii lpp* length mutants demonstrated that the elongated Lpp+21 variant enhanced the efficacy of OutB attachment to PG, whereas the truncated LppΔ21 variant diminished it. In this context, the length of OutB (OutBΔ22, OutB, or OutB+21) did not affect these trends. It is tempting to suggest that such alterations in the extent of OutB linking to PG are caused by displacements of the PG layer in the periplasm of *D. dadantii lppΔ21* and *lpp* + *21* mutants. Indeed, previous studies have shown that in *lpp* + *21* mutants of *E. coli* and *S. enterica*, the PG layer was closer to the IM than in the wild-type strain^40,41^ and exhibited a broader and more diffuse morphology^41^. Such a diffuse architecture of PG layer may explain the enhanced diffusion of free forms of OutB through the PG mesh of the *D. dadantii lpp* + *21* mutant (Fig. 3C).

Another reported consequence of the increased periplasm width in the *E. coli lpp* + *21* mutant was the inactivation of the Rcs signaling pathway^39^. In this cellular context, the size of the RcsF sensor lipoprotein has become insufficient to establish contact with the IM sensor IgaA^39^. The Rcs signaling pathway is known to activate the expression of genes involved in capsule synthesis (*cps*) and cell division (*ftsZ*), while repressing the transcription of flagellar genes^53,54^. Accordingly, the phenotypes of the *D. dadantii lppΔ21* mutant (mucoid, small, and non-motile cells) (Figs. 3B, S6D, E and S7) are indicative of a constitutively activated Rcs system. It seems plausible that, due to the diminished periplasmic width in the *D. dadantii lppΔ21*, the OM-located RcsF remains in continuous contact with the IM sensor IgaA, thereby activating the Rcs cascade. It is likely that the elevated exopolysaccharide production is responsible for the resistance of *D. dadantii lppΔ21* to SDS, despite the apparent alterations in cell morphology (Figs. 3B and S8). It is noteworthy that a search for vancomycin-resistant mutants of *E. coli* has resulted in the isolation of a spontaneous in-frame *lpp*Δ*21* strain with a fourfold increased resistance to the antibiotic^55^. It seems plausible that an increased production of exopolysaccharides by the *lpp*Δ*21* mutant could provide generalized protection against desiccation and external agents^56^. In contrast, *lpp* + *21* mutants showed an increased susceptibility to SDS (Figs. 3B and S6D) and to vancomycin^42^.

Site-directed mutagenesis of the Lpp-like box of OutB showed that it can tolerate substantial residue substitutions. Markedly, the presence of a double positive charge (Lys-Lys) at the C-terminus is not mandatory. The OutB_K220tga variant lacking the C-terminal Lys^220^ was attached to PG by the remaining penultimate Lys^219^, which became the C-terminal residue (Fig. 1D). Indeed, the truncated Lpp-box of OutB_K220tga (RTTK) is quite similar to those of the Lpp orthologs from *Photobacterium*, *Vibrio,* and *Zobellella*, namely S/RYTK^21^. Interestingly, in *E. coli*, over 20% of periplasmic proteins and lipoproteins possess a C-terminal Lys residue^57^. Taken together with the apparent absence of a well-conserved Lpp-like consensus, this led us to suggest that some other periplasmic and/or membrane proteins may also be covalently attached to the PG in *D. dadantii* and probably in other proteobacteria.

## Methods

### Strains, plasmids, growth conditions, and mutagenesis

The bacterial strains and plasmids used in this study are listed in Table S2. Bacteria were grown in lysogeny broth (LB) at 28 °C with shaking at 120 rpm. When necessary, glycerol was added at 0.2% and antibiotics were supplemented at follows: ampicillin, 50 mg/L; kanamycin, 50 mg/L, and chloramphenicol, 25 mg/L. For PG extraction, bacteria were typically grown to late exponential phase until an optical density at 600 nm (OD_600_) of about 2.0 was reached. For production of OutB variants, *D. dadantii outB* A5654 cells carrying the BS plasmid with an *outB* mutant gene of interest were grown in LB supplemented with 0.2% glycerol and 50 mg/L ampicillin at 28 °C for 4 h to an OD_600_ of ~0.5, induced with 1 mM isopropyl-*β*-D-thiogalactopyranoside (IPTG), and grown for a further 5 h. The expression of the plasmid-borne *lpp* and *ldt* genes was not induced by the addition of any inducer. In this order, an appropriate *D. dadantii* mutant strain carrying the pGEM-T plasmid with the gene of interest was grown in LB supplemented with 0.2% glycerol and 50 mg/L ampicillin at 28 °C for 12 h to late exponential phase (OD_600_~2.0).

Site-directed mutagenesis was carried out using the PrimeSTAR Max DNA Polymerase (TaKaRa Bio Inc., Japan) with the primers listed in Table S3. The sequences of mutant and amplified genes were checked (Eurofins Genomics, Germany, or Microsynth AG, Switzerland). *D. dadantii* mutant strains carrying chromosomal *ldt* or *lpp* mutant alleles were constructed by homologous recombination followed by de novo transduction of mutant alleles with phage phi-EC2^58^.

### PG purification for western blotting analysis

For PG extraction, bacteria were grown as specified above. Cells (~5 × 10^10^) were collected by centrifugation (7000 × *g*, 5 min), resuspended in 2 mL of 50 mM Tris-HCl, 1 mM EDTA (TE) buffer, then supplemented with 2.5 mL of 10% SDS, boiled at 100°C for 1 h, and stored overnight at 30 °C. PG was pelleted by centrifugation (100,000 × *g*, 2 h), resuspended in 4 mL of 2% SDS, boiled for 30 min, and pelleted again (100,000 × *g*, 1 h). Next, PG was resuspended in TE, boiled for 30 min, and collected by centrifugation (20,000 × *g*, 1 h). This washing step was repeated thrice, and PG was resuspended in 0.5 mL of TE. 80 µL of the PG sample was digested for 3 h at 37 °C with 4 µg of lysozyme (Sigma–Aldrich) and then, half of the sample was completed with 2 mM MgSO_4_, 0.1 mM ZnSO_4_, and 1 µg of PGRP amidase from *Sitophilus zeamais*^37^ (kindly provided by Professor Pedro Da Silva, INSA Lyon), and digested for an additional 1 h at 37 °C. The PG samples were loaded on SDS-PAGE immediately after digestion.

### SDS-PAGE and immunoblotting

PG samples digested with lysozyme and PGRP amidase were supplemented with Laemmli loading buffer, boiled for 7 min, and undigested PG material was pelleted at 10,000 × *g* for 10 s. The samples were separated in 10% tris-glycine (for OutB) or 15% tris-tricine (for Lpp) SDS-polyacrylamide gel and transferred to Roti PVDF 0.45 µm membrane (Roth). Blots were probed with rabbit polyclonal antibodies raised against OutB^35^ or *D. dadantii* Lpp (this work) at 1:1500 dilution followed by secondary anti-rabbit goat IgG conjugated to peroxidase (Sigma-Aldrich, St. Louis, USA) at 1:20,000 dilution. Glycoproteins containing *N*-acetylglucosamine (GlcNAc) residues were detected with wheat germ agglutinin, WGA conjugated to peroxidase at 1:25,000 (Sigma–Aldrich). Chemiluminescence signals generated with Immobilon substrate (Millipore, Burlington, USA) were detected using Fusion FX imaging system (Vilber Lourmat, Marne-la-Vallee, France) or Hyperfilm (Cytiva Sweden AB, Uppsala, Sweden) and quantified with EvolutionCapt Edge (Vilber Lourmat) or with Fiji software^59^.

### Light microscopy

For microscopic observations, bacteria were grown in 10 mL of LB at 28 °C for 5 h (reaching ca. the mid-exponential growth phase, DO_600_ of 0.4–0.6 depending on strain) with shaking at 120 rpm. Cells were collected by centrifugation (6000 × *g*, 5 min) and washed one time in phosphate buffer saline pH 7.2 (Sigma–Aldrich) and directly processed with microscopy as described below.

Photos were taken using the LAS AF program with a Leica DM6000B microscope. Magnification used ×40, time of exposure: 30,178 ms, Gain: 1,7, Light intensity: 75, DIC Prism: D, Condenser Prism: K3, and DIC Finetunning: −478. Bacteria were measured in the ImageJ program^59^.

### Transmission electron microscopy (TEM)

The cells were adsorbed onto carbon grids (EMSdiasum) and stained with 1.5% uranyl acetate (BDH Chemicals Ltd, Pool Dorset, UK) for contrast enhancement. The samples were analyzed with a Tecnai Spirit BioTWIN transmission electron microscope at 120 kV. Measurements were made with Radius EM Imaging Software (Emsis GmbH, Münster, Germany).

### Scanning electron microscopy (SEM)

Bacterial cells were fixed overnight in 2.5% glutaraldehyde, postfixed in 1% osmium tetroxide (Agar), and gradually dehydrated in ethanol. Bacteria were mounted onto SEM stubs and coated with gold using a sputter coater (SPI Supplies, West Chester, USA). The samples were examined and photographed using a Prisma E Scanning Electron Microscope (Thermo Fisher Scientific, Waltham, USA).

### Mass spectrometry analyses of peptidoglycan structure

For MS analysis, PG was extracted by the hot SDS procedure, and proteins were removed with trypsin. Specifically, bacterial cells from 200 mL of culture grown to late exponential phase (OD_600_ of about 2.0) were resuspended in 5 mL of water, completed with 5 mL of 10% SDS and incubated with mixing at 100 °C fo 1 h. PG was collected by centrifugation (95,000 × *g* for 1 h at 25 °C) and washed five times with 25 mL of water (95,000 × *g* for 30 min at 25 °C). PG was then resuspended in 1 mL of phosphate buffer (20 mm, pH 7.8) and treated for 6 h at 37 °C with trypsin at 200 μg/ml. Next, PG was washed three times with 25 mL of water, resuspended in 0.5 mL of phosphate buffer (25 mm, pH 6.0) supplemented with 0.1 mm MgCl_2_ and digested with mutanolysin (200 μg/mL; Sigma–Aldrich) and lysozyme (200 μg/mL; Sigma-Aldrich) at 37 °C for 12 h to obtain soluble disaccharide-peptide fragments. MurNAc residues were then reduced to *N*-acetylmuramitol^60^. In this order, 200 µl of borate buffer (250 mM, pH 9.0) was added to 200 µl of the solution of disaccharide peptides. Two mg of sodium borohydride were added to the resulting solution followed by incubation for 20 min at room temperature. The pH of the solution was adjusted to 4.0 with 20% orthophosphoric acid. The resulting muropeptides were separated by *rp*HPLC on a C_18_ column (Hypersil GOLD aQ 250 ×4.6, 3 µm; Thermo Fisher Scientific) at a flow rate of 1 mL/min. A linear gradient (0–100%) was applied between 11.1 min and 105.2 min at 20 °C (buffer A: 0.1% TFA; buffer B: 0.1% TFA, 20% acetonitrile; v/v). Muropeptides were detected by absorbance at 205 nm. One mL fractions were automatically collected with a fraction collector, lyophilized, solubilized in 20 µL of water, and stored at −20 °C. Mass spectra were obtained by injecting an aliquot of the fractions (5 μL) into the mass spectrometer (Maxis II ETD, Bruker, France) at a flow rate of 0.1 mL/min (50% acetonitrile, 50% water; v/v acidified with 0.1% formic acid; v/v). Spectra were acquired in the positive mode with a capillary voltage of 3500 V, an *m*/*z* scan range was from 300 to 1850 at a speed of 2 Hz. Transfer time stepping was performed with RF values 400 and 1200 Vp-p, transfer times 30 and 90 μs, and timing 50 and 50%. MS/MS spectra were obtained using a collision energy of 50 eV in the *m*/*z* range of 150–1000 for muropeptide monomers and 150–2000 for muropeptide dimers with isolation width of 1. The applied collision energy varied from 77 to 100% of the 50 eV setting with timings of 33% and 67%, respectively^61^.

### Statistics and reproducibility

Statistical analysis was performed with PRISM software (version 10.4.1. GraphPad). For microscopy analysis of cell length, 100 cell micrographs were randomly selected for each strain. All the collected values, including outliers, were taken for analysis. The data were analyzed using two-sample *t-*test by comparing each mutant to the wild-type strain. The data are presented as mean and SEM (standard error of the mean). Dots represent the length value of each cell. Signal intensity of Western blots was quantified with EvolutionCapt Edge (Vilber Lourmat) or with Fiji software. The data from at least three technical replicates of two biological experiments were collected and analyzed using a two-sample Mann–Whitney test by comparing the values of mutant to wild type considered as 100%. Graphs were generated using PRISM software.

### Reporting summary

Further information on research design is available in the Nature Portfolio Reporting Summary linked to this article.

## Supplementary information

Supplementary Material

Description of Additional Supplementary Files

Supplementary Data 1

Reporting summary

## Data Availability

The data supporting the findings of this study are available within the paper and its Supplementary Information that includes Figs. S1–S15 and Tables S1–S3 and Supplementary Methods. Uncropped and unedited blot and gel are presented as Supplementary Fig. S15. Source data for statistical analyses are provided in Supplementary Data 1. The raw data of MS analyses have been deposited in Zenodo open repository under accession code https://zenodo.org/records/15691255.
