# Supplementary material for: An inner membrane protein is covalently attached to peptidoglycan in the γ-proteobacterium Dickeya dadantii
Source: Commun Biol. 2025 Jul 18;8:1071. doi: 10.1038/s42003-025-08488-9 (PMC12274547; doi:10.1038/s42003-025-08488-9)
Supplement: Supplementary file 1 — Supplementary Material [file 42003_2025_8488_MOESM1_ESM.pdf]

Supplementary Information for

An inner membrane protein is covalently attached to peptidoglycan in the γ-proteobacterium *Dickeya dadantii*

Xavier NICOLAI[1], Yucheng LIANG[2], Florence RUAUDEL[1], Magdalena NARAJCZYK[3], Robert CZAJKOWSKI[4], Filippo Rusconi[2,5], Michel ARTHUR[2] and Vladimir E. SHEVCHIK[1*]

Corresponding author

Vladimir E. Shevchik vladimir.shevchik@insa-lyon.fr

This file includes

Figures S1 to S15,

Tables S1, S2 and S3,

Supplementary Methods,

Supplementary References

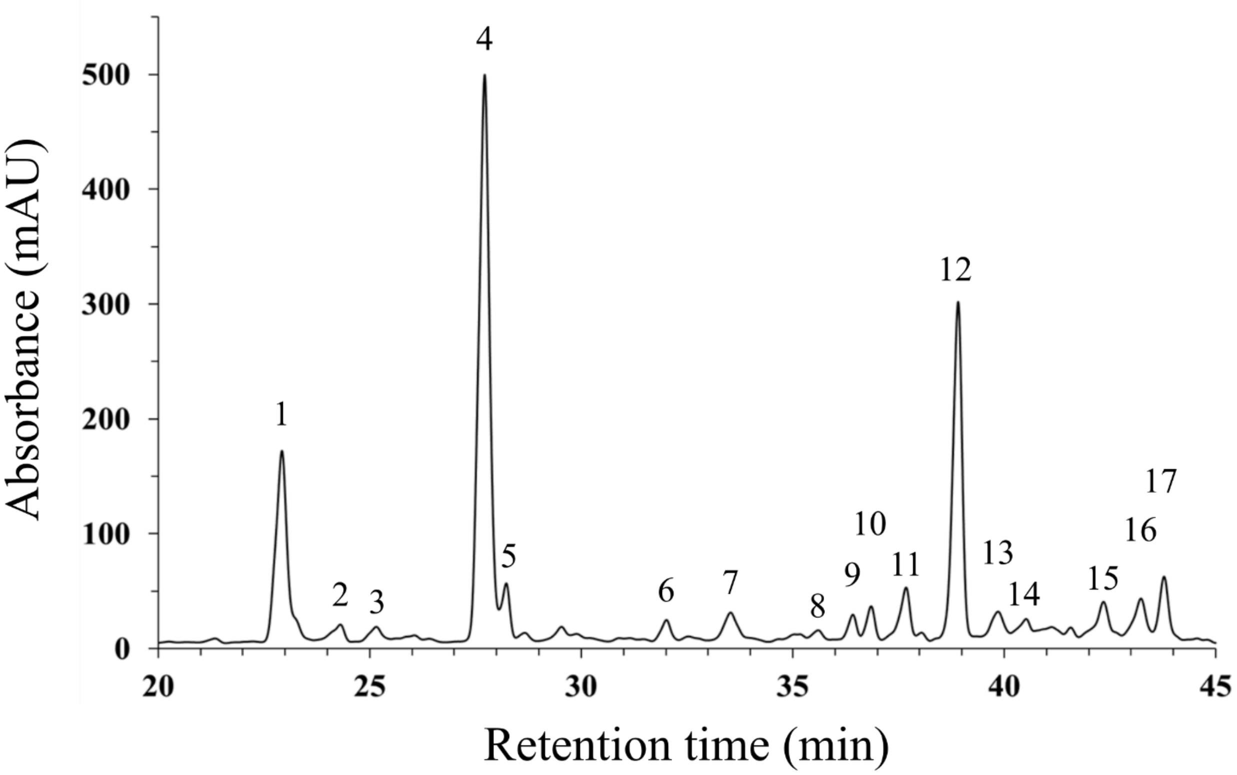

Figure S1. Chromatogram of the *rp*HPLC separation of the muropeptides from *Dickeya dadantii* 3937*.*

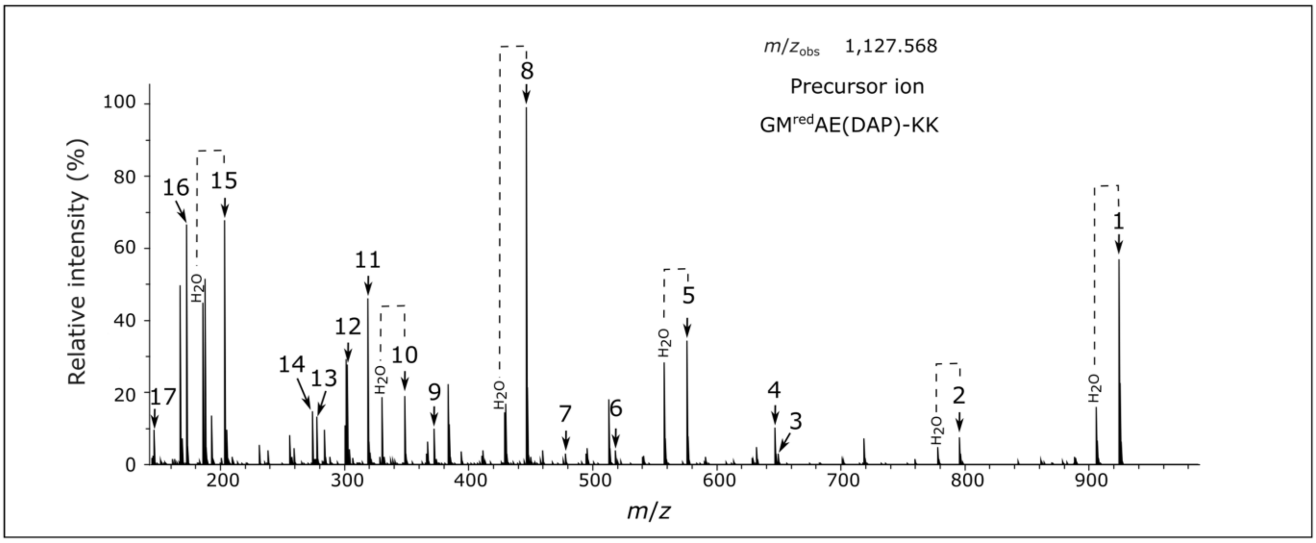

	1	2	3	4	5	6	7	8
Product ion	M^{red}AE(DAP)-KK	M^{red}AE(DAP)-K	M^{red}AE(DAP)	AE(DAP)-KK	E(DAP)-KK	AE(DAP)-K	M^{red}AE	(DAP)-KK
m/z_{obs}	924.488	796.392	650.289	647.373	576.335	519.279	478.294	447.294
M_{obs}	923.480	795.385	649.281	646.365	575.327	518.271	477.286	446.286
M_{cal}	923.481	795.386	649.281	646.365	575.328	518.270	477.285	446.285

	9	10	11	12	13	14	15	16	17
Product ion	AE(DAP)	M^{red}A	(DAP)-K	E(DAP)	M^{red}	KK	G	(DAP)	K
m/z_{obs}	373.171	349.160	319.198	302.134	278.125	275.209	204.089	173.094	147.115
M_{obs}	372.163	348.152	318.191	301.126	277.117	274.201	203.081	172.086	146.107
M_{cal}	372.164	348.153	318.190	301.127	277.116	274.200	203.079	172.085	146.106

Figure S2. Tandem mass spectrometry analysis of the Tri-KK monomer. Fragmentation was performed on the precursor ion [GM^{red}AE(DAP)-KK], *m/z* 1,127.568; M_{obs} = 1,126.560; M_{cal} = 1,126.561. Abbreviations: M^{red}, reduced MurNAc; A, L-Ala; E, D-Glu; DAP, diaminopimelic acid, K, L-Lys.

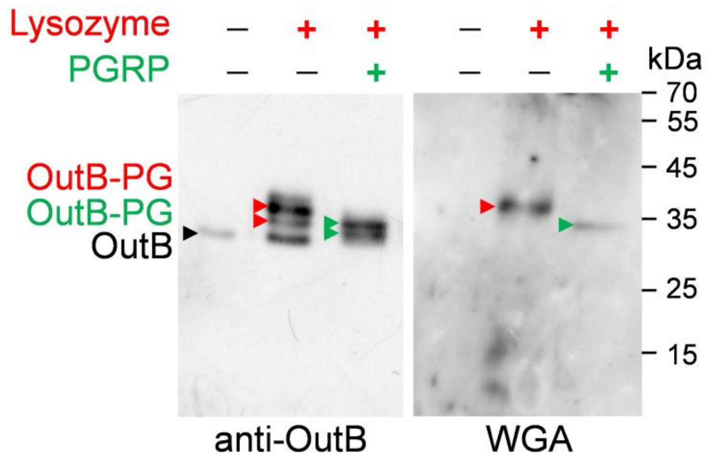

Figure S3. Western blot analysis of OutB-PG species linked to muropeptides in *D. dadantii lpp* mutant. *D. dadantii Δlpp* expressing *outB* from BS plasmid were grown in LB supplemented with 0.2% glycerol and 50 mg/L ampicillin at 28°C for 16 h to late-exponential phase (OD_{600}~2.0) without addition of IPTG. PG was extracted and digested or not with lysozyme or PGRP amidase and analyzed by Western blot with anti-OutB or WGA. The positions of OutB-PG species generated by lysozyme and PGRP amidase are indicated with red and green arrows, respectively. The position of "free" form of OutB is shown with black arrow.

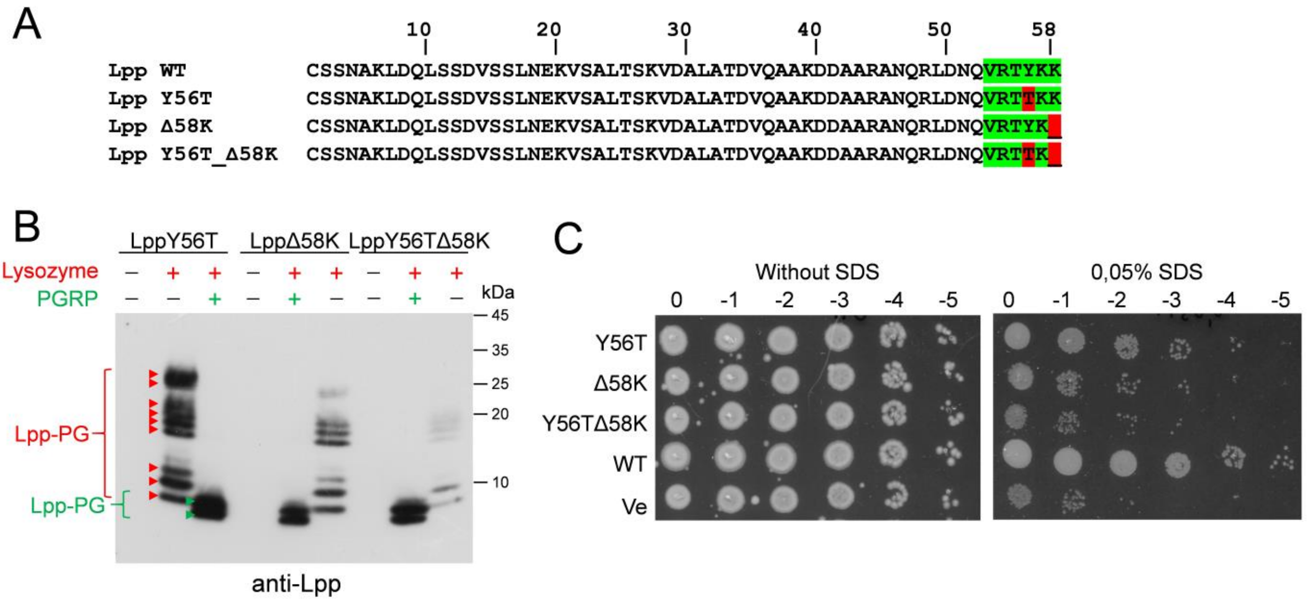

Figure S4. Mutagenesis of the Lpp box of Lpp$_{Dd}$. (**A**) Sequence alignment of *D. dadantii* Lpp mutants. The Lpp-box is highlighted in green and the substituted residues are shown in red. (**B**) PG from *D. dadantii lpp* mutant strain producing the indicated Lpp variants from pGEM-T_Lpp plasmid was digested or not with lysozyme and PGRP amidase and probed by Western blot with anti-Lpp antibodies. Positions of the generated Lpp-PG species are indicated with red and green arrows, respectively. **Please, note that 50-fold more PG material was loaded for this Western blot than on that with the wild-type Lpp shown in Fig. 1B.** (**C**) SDS susceptibility assay with the Lpp variants. Overnight cultures of *D. dadantii lpp* ectopically expressing indicated Lpp variants from pGEM-T_Lpp plasmid were serially diluted and plated onto LB agar, whether or not containing 0.05% SDS and incubated for 24 h at 28°C. Ve indicates empty vector.

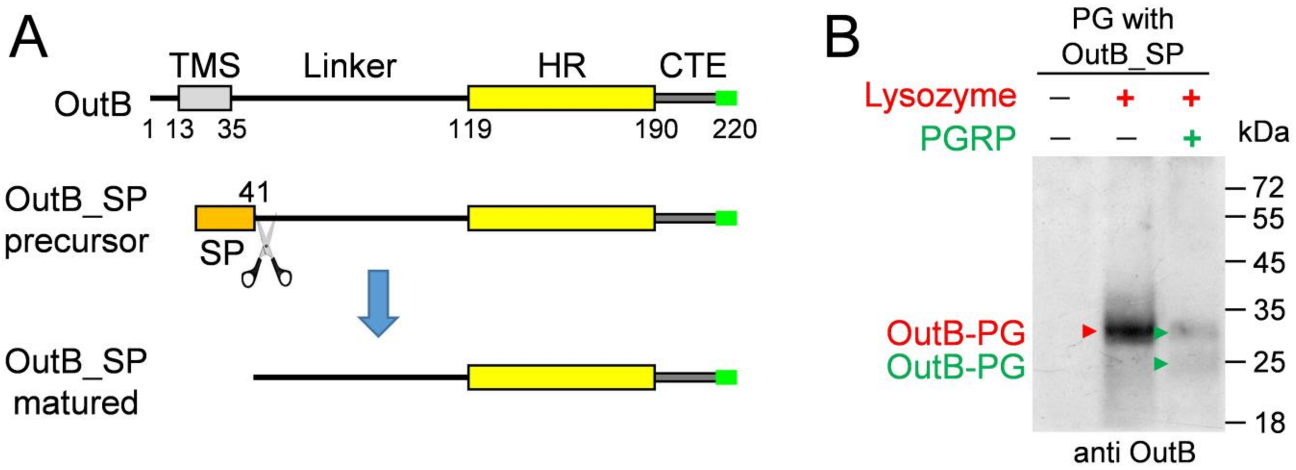

Figure S5. The inner membrane anchoring is not essential for attachment of OutB to PG. (**A**) Schematic of OutB_PS variant carrying a cleavable signal peptide (SP) originated from pET-20b in place of the native TMS. (**B**) Western blot analysis of PG purified from *D. dadantii* expressing OutB_PS. PG was digested or not with lysozyme and PGRP amidase and probed with anti-OutB antibodies. Generated OutB-PG adducts are indicated with red and green arrows, respectively.

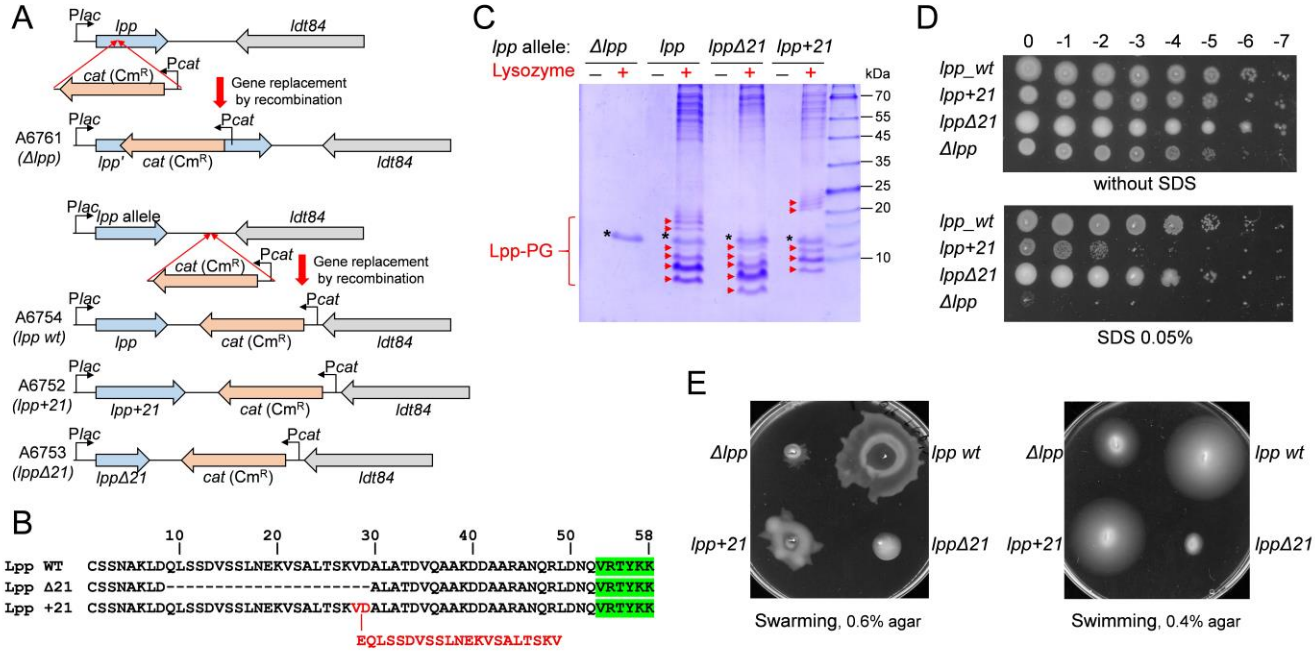

Figure S6. Construction and phenotypic assessment of *D. dadantii lpp* mutant strains. (**A**) Construction of *D. dadantii lpp* mutant strains. The mutated *lpp* alleles were introduced into the *D. dadantii* chromosome in place of the wild-type *lpp* gene by homologous recombination. The *cat* (Cm^R) gene was used as a selective marker for *de novo* transductions of the mutated *lpp* alleles into the *D. dadantii* wild type strain. (**B**) Sequence alignment of the Lpp length variants. The additional 21-residue sequence of Lpp+21 and its insertion site are shown in red letters. (**C**) PG from *D. dadantii* strains carrying the indicated (on top) *lpp* alleles was purified, digested or not with lysozyme and analyzed by SDS-PAGE. Lpp-PG species are shown with red arrows and lysozyme is noted with an asterisk. (**D**) SDS susceptibility assay. Overnight cultures were serially diluted and plated onto LB agar, whether or not containing 0.05% SDS and incubated for 24 h at 28°C. (**E**) Swarming (left) and swimming (right) motility assays. ~10^6 of overnight grown cells were deposited into the soft LB agar (0.6 or 0.4%, respectively) and cultivated at 28°C for 14 h.

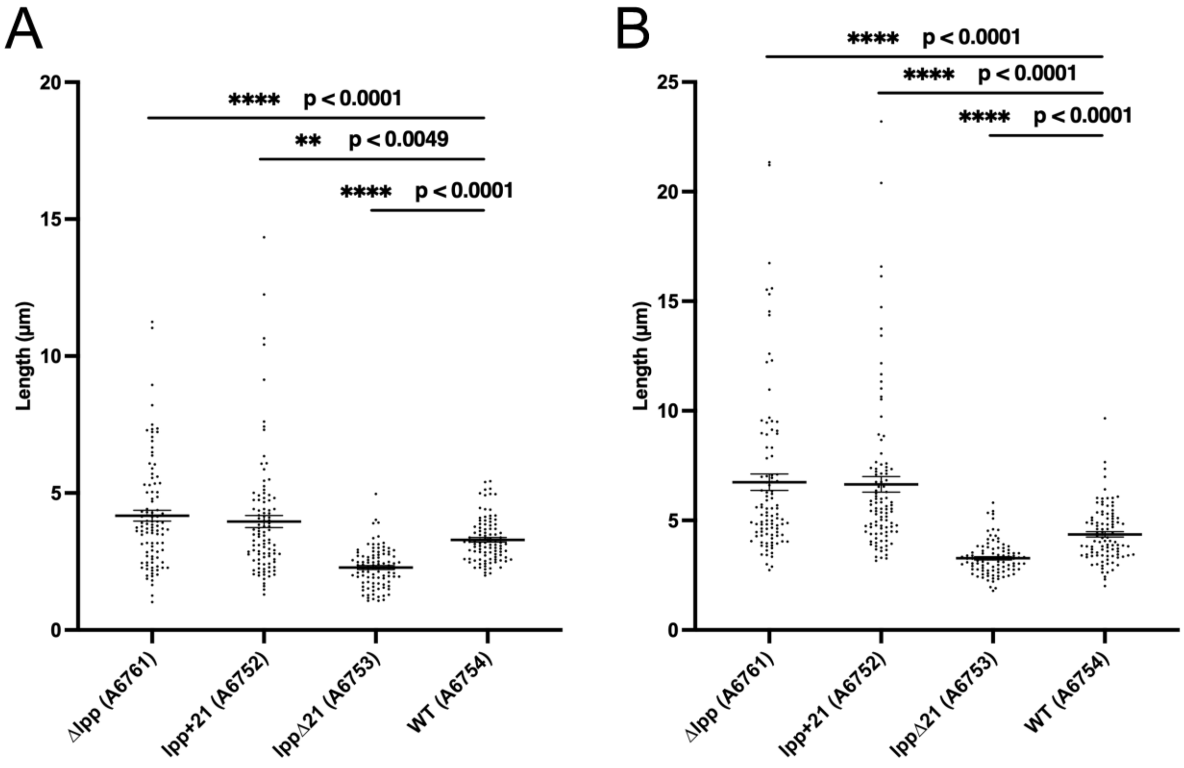

Figure S7. Length of *D. dadantii lpp* mutant cells observed in negative stain EM micrographs (A) and in light microscopy (Nomarski interference contrast) (B). For each strain, 100 cell micrographs were randomly selected. All the collected values, including outliers, were taken for analysis. The data were analyzed with PRISM software using two-sample *t*-test by comparing each mutant to the wild-type strain. Dots represent the length value of each cell. Mean and SEM are shown with horizontal bars. **** and ** denote statistically significant differences between mean values compared to the WT strain A6754, with *P* values <0,0001 and <0,0049, respectively. Source data are provided in Supplementary Data 1.

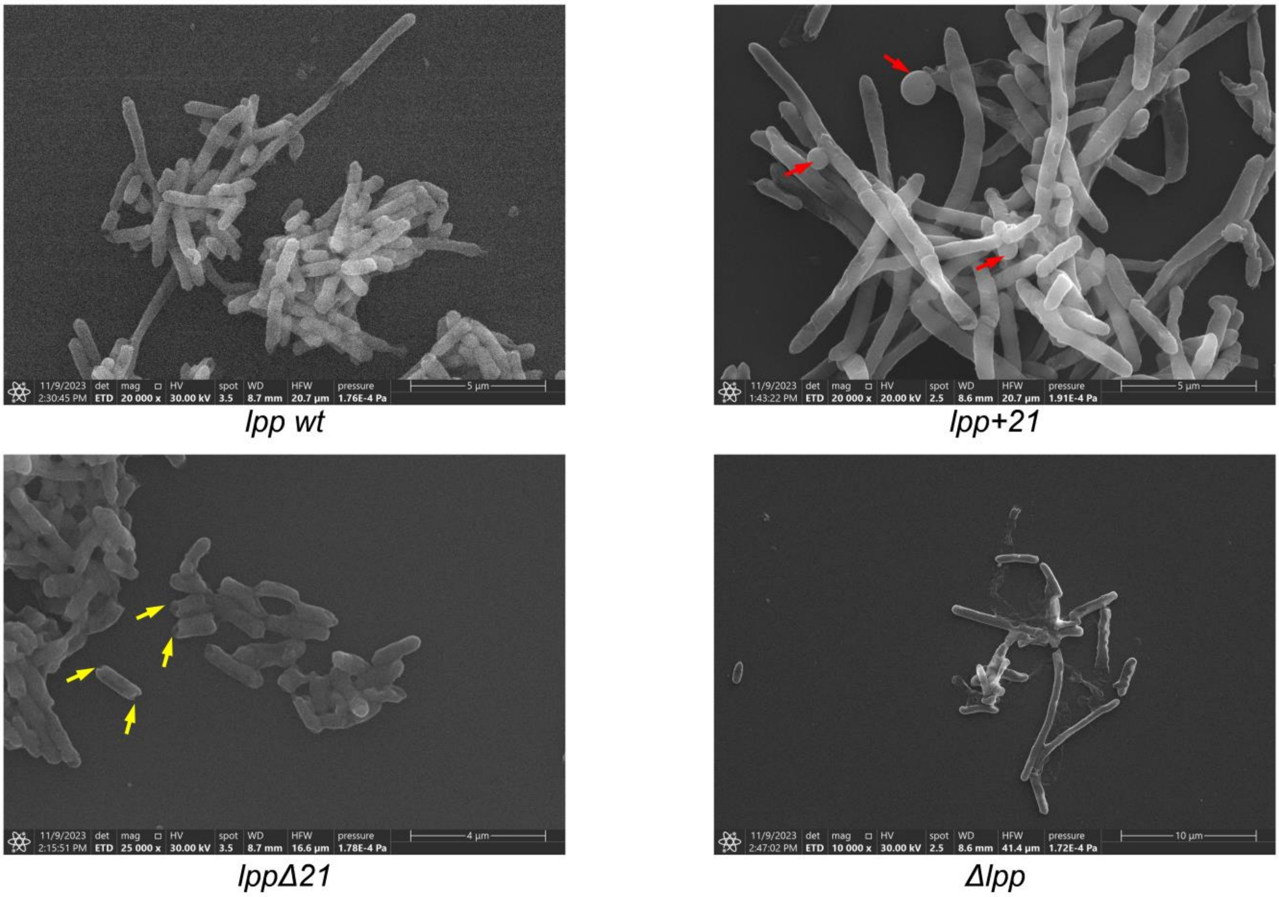

Figure S8. Scanning Electron Microscopy of the indicated *D. dadantii lpp* mutant strains. The blebs in the *lpp+21* strain are shown with red arrows and the cavities at the cell poles of the *lppΔ21* mutant are indicated with yellow arrows.

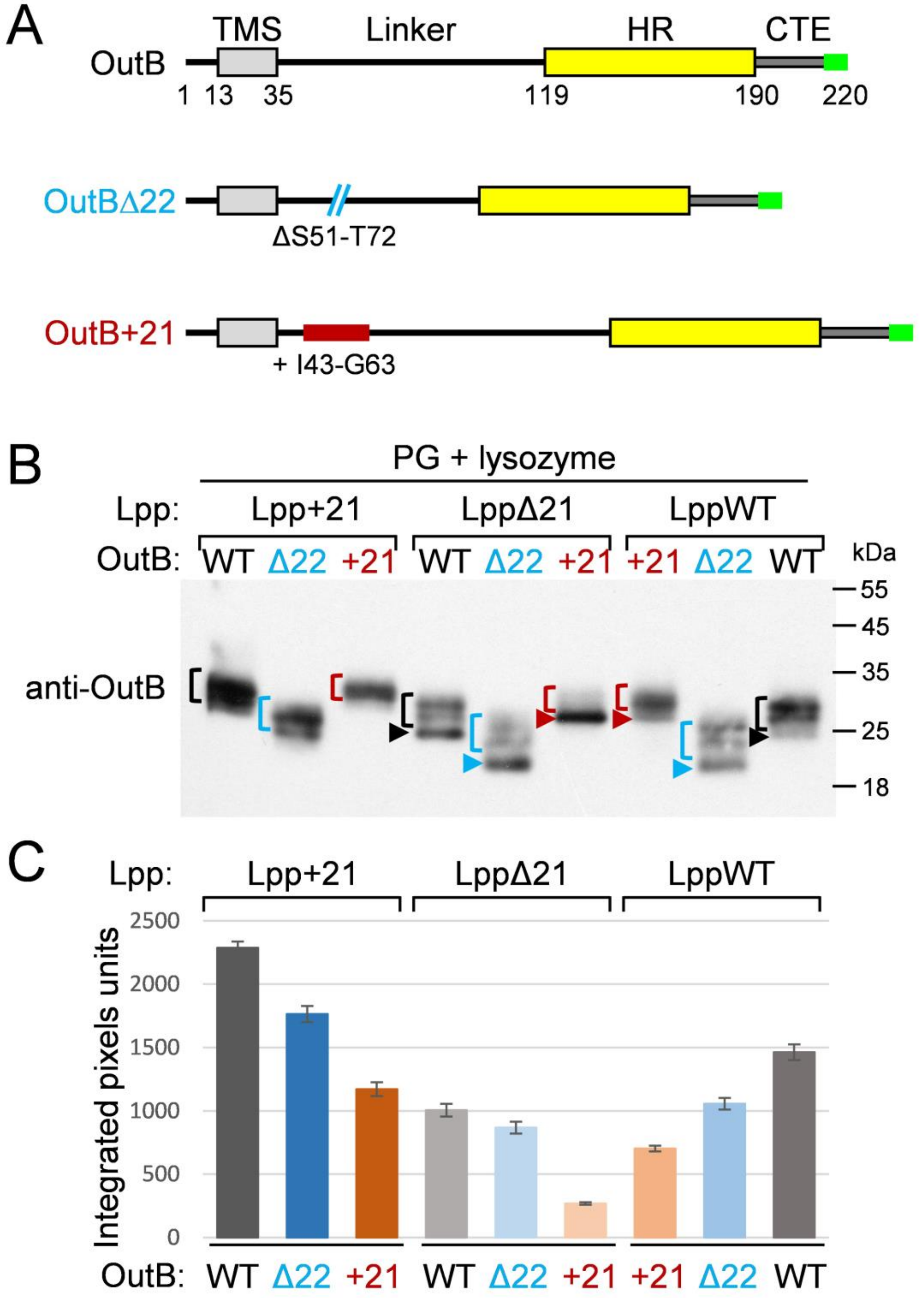

Figure S9. Lengthening of Lpp improves attachment of OutB length variants to PG. (**A**) Schematic of OutB length variants. The regions deleted (Ser^{51} to Thr^{72}) or inserted (Ile^{43} to Gly^{63}) in OutBΔ22 and OutB+21, respectively, are indicated. (**B**) Representative immunoblotting analysis of PG purified from *D. dadantii* WT, *lppΔ21*, and *lpp+21* strains producing either OutBwt, OutBΔ22, or OutB+21 (nine combinations indicated on the top of panel). Only lysozyme-digested samples are shown. “Free” forms of OutBwt, OutBΔ22 and OutB+21 are indicated with black, blue and dark red arrowheads, respectively. OutB-PG species linked to muropeptides are shown with

brackets of the same colors. (**C**) Muropeptide-linked specie of each OutB variant (shown with brackets in panel B) were quantified with EvolutionCapt Edge Software (Viber Lourmat) according to the pixel intensity and area of each protein band. The data are from the replicate shown in panel B. The bar heights show the relative amount of muropeptide-linked species for each OutB-length variant in each *D. dadantii lpp*-length mutant line (as they are shown with brackets in panel B). The bars are positioned below the corresponding gel lines in panel B.

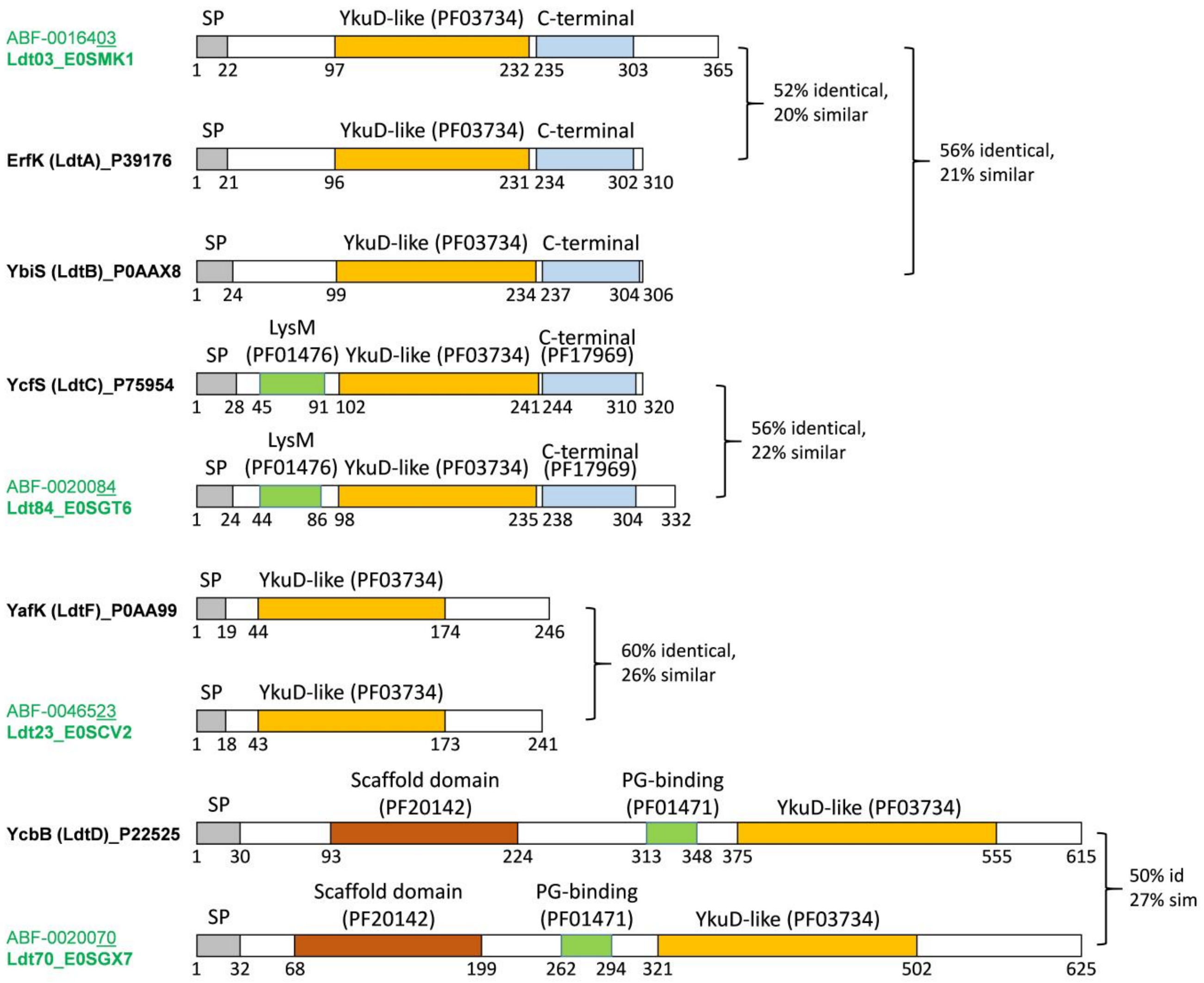

Figure S10. Domain organization of L,D-transpeptidases of *D. dadantii* compared with those of *E. coli*. Domain occurrence and their positions are from InterPro (10.1093/nar/gkac993). The names of Ldts are followed with their UniProtKB codes. The names of *D. dadantii* Ldts are in green and those of *E. coli* are in black. The identity and similarity levels of orthologous Ldts are indicated.

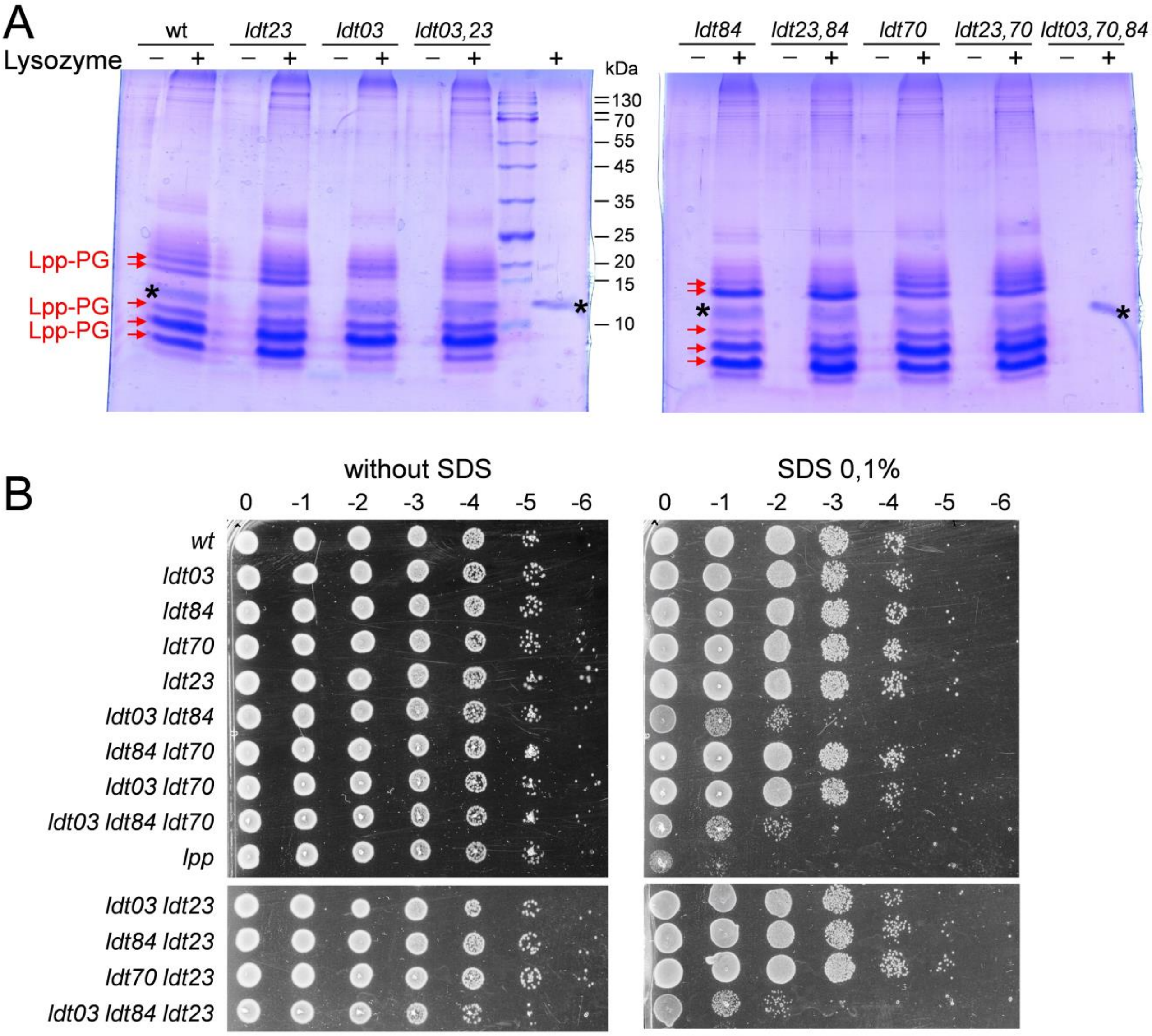

Figure S11. Characterization of *D. dadantii ldt* mutant strains. (**A**) SDS-PAGE analysis of the protein content of PG purified from *D. dadantii ldt* mutant strains. PG was digested or not with lysozyme. Lpp-PG adducts generated by lysozyme are indicated with red arrows and lysozyme position is shown with an asterisk. (**B**) SDS susceptibility assay with *D. dadantii ldt* mutant strains. Overnight cultures were serially diluted and plated onto LB agar, whether or not containing 0.1 % SDS and incubated for 24 h at 28°C.

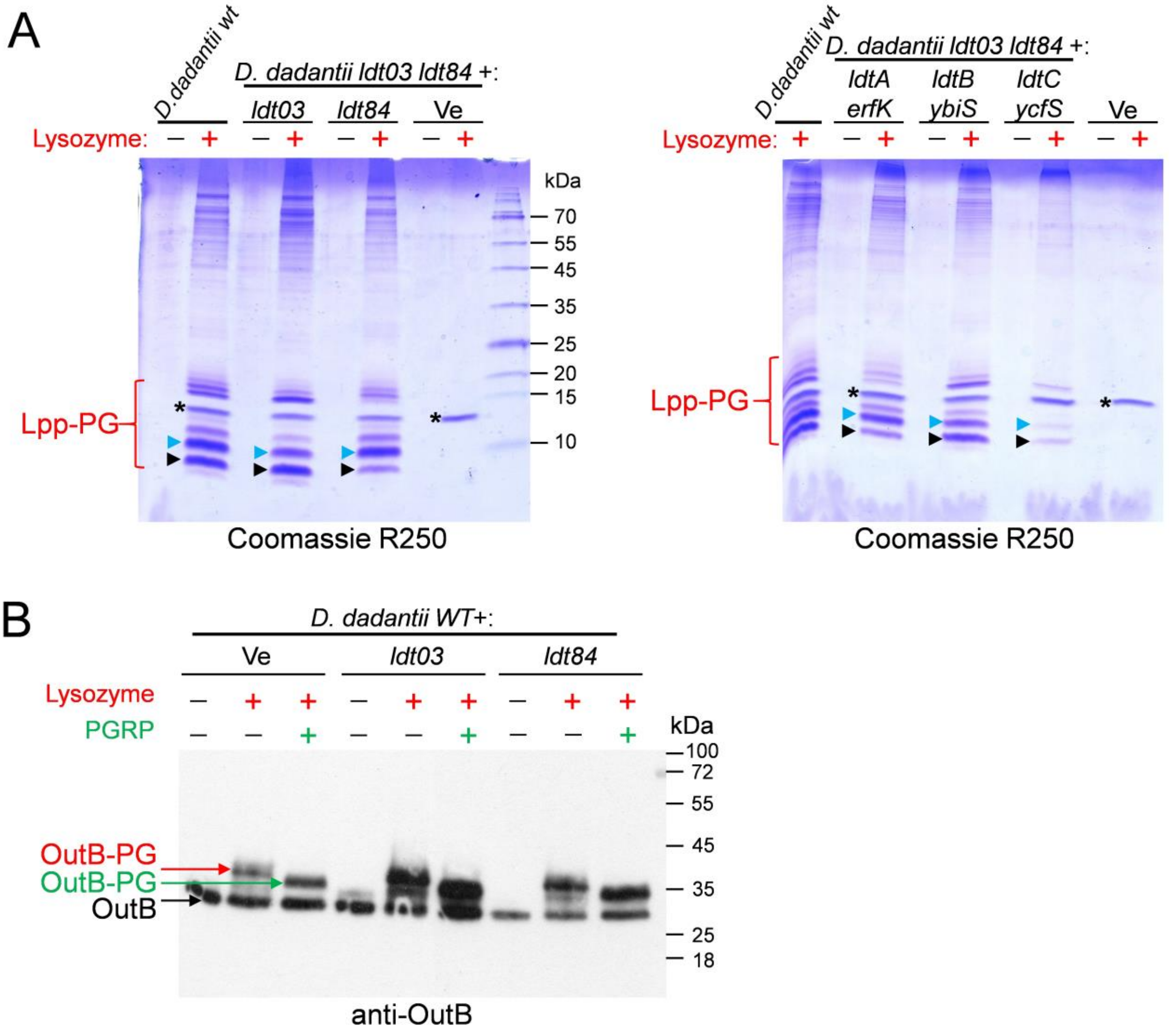

Figure S12. Different L,D-transpeptidases generate dissimilar muropeptide linked patterns of Lpp and OutB. (**A**) Comparative analysis of Lpp-muropeptide patterns generated by Ldt03 and Ldt84 of *D. dadantii* (left panel) and LdtA, LdtB and LdtC of *E. coli* (right panel). PG from *D. dadantii* wild type or *ldt03 ldt84* double mutant ectopically expressing one of the mentioned *ldt* genes or empty pGEM-T vector (Ve) was digested or not with lysozyme and analyzed by SDS-PAGE. Lpp-PG adducts are shown with red bracket. Blue and black arrows indicate Lpp adducts linked to dimeric and monomeric muropeptides, respectively. (**B**) Ectopic expression of *ldt03* or *ldt84* increased covalent attachment of OutB to PG in the *D. dadantii* WT strain. PG from *D. dadantii* WT carrying either empty plasmid (Ve) or that with *ldt03* or *ldt84* was digested or not with lysozyme and PGRP and analyzed by Western blot with anti-OutB antibodies. The positions of “free” OutB and OutB-PG adducts generated by lysozyme and PGRP are indicated with black, red and green arrows, respectively.

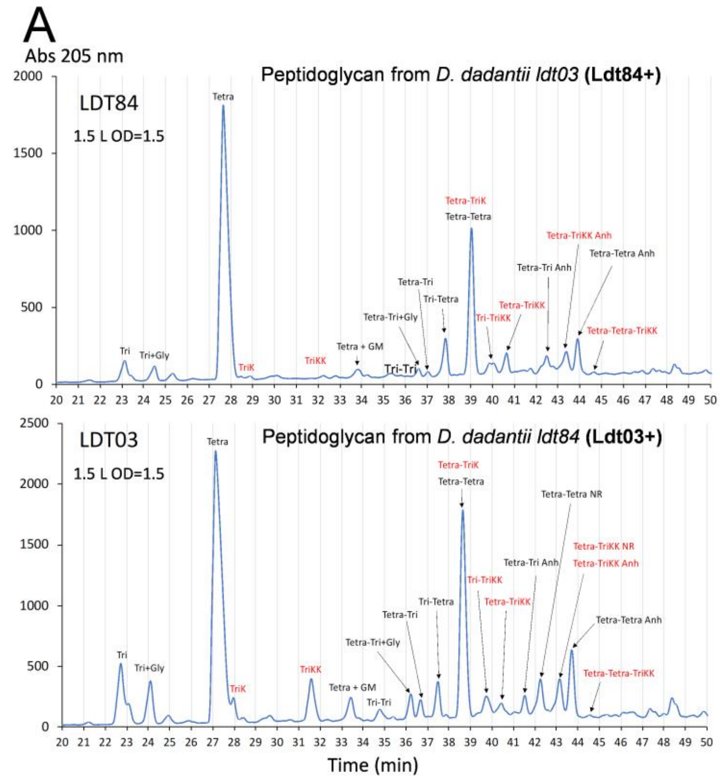

B

Relative abundance of muropeptides resulting from the PG-anchoring of Lpp

	Relative intensity (%) in strains lacking *ldt03* or *ldt84*			
	ldt03 (Ldt84+)		*ldt84* (Ldt03+)	
**Replicate**	**Tri→KK**	**Tetra→Tri→KK**	**Tri→KK**	**Tetra→Tri→KK**
1	16.7	83.3	86.0	14.0
2	33.4	66.6	73.6	26.4
3	33.8	66.2	77.8	22.2
Mean	28.0	72.0	79.1	20.9
Standard deviation	9.8	9.8	6.3	6.3

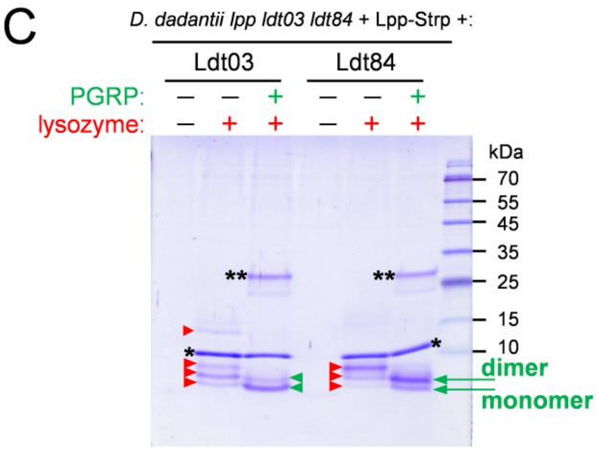

Figure S13. Ldt03 and Ldt84 preferentially attach Lpp to monomeric and dimeric peptide stems, respectively. (**A**) *rp*HPLC profile analysis of muropeptides generated from *D. dadantii ldt03* (Ldt84+, upper panel) and *D. dadantii ldt84* (Ldt03+, lower panel) mutants. Bacteria were grown until late exponential phase (DO_{600} ~2.0) and PG was extracted by the hot SDS-procedure, digested with pronase, trypsin, and muramidases. The resulting muropeptides were reduced with $NaBH_4$, separated by *rp*HPLC and identified by MS. The names of muropeptides plotted on the pics correspond to those in Fig. S2. Muropeptides bearing Lys or Lys-Lys are in red. (**B**) Relative abundance of the Tri→KK monomer and Tetra→Tri→KK 4→3 cross-linked dimer containing the Lys-Lys motif in the PG extracted either from *D. dadantii ldt03* carrying a chromosomic copy of the *ldt84* gene (Ldt84) or from *D. dadantii ldt84* carrying a chromosomic copy of *ldt03* (Ldt03). Values are the ion current intensity of corresponding analytes as detected by mass spectrometry. The respective muropeptide patterns are shown in panel A. (**C**) Comparative assessment of Lpp-muropeptide patterns generated by Ldt03 and Ldt84. PG from *D. dadantii lpp ldt03 ldt84* triple mutant ectopically expressing *lpp-Strep* (pGEM-T) together with either *ldt03* or *ldt84* (pBS-Km) was digested or not with lysozyme and PGRP amidase and analyzed by SDS-PAGE. Lpp-Strep-PG species linked to muropeptides generated by lysozyme and PGRP are indicated with red and green arrowheads, respectively.

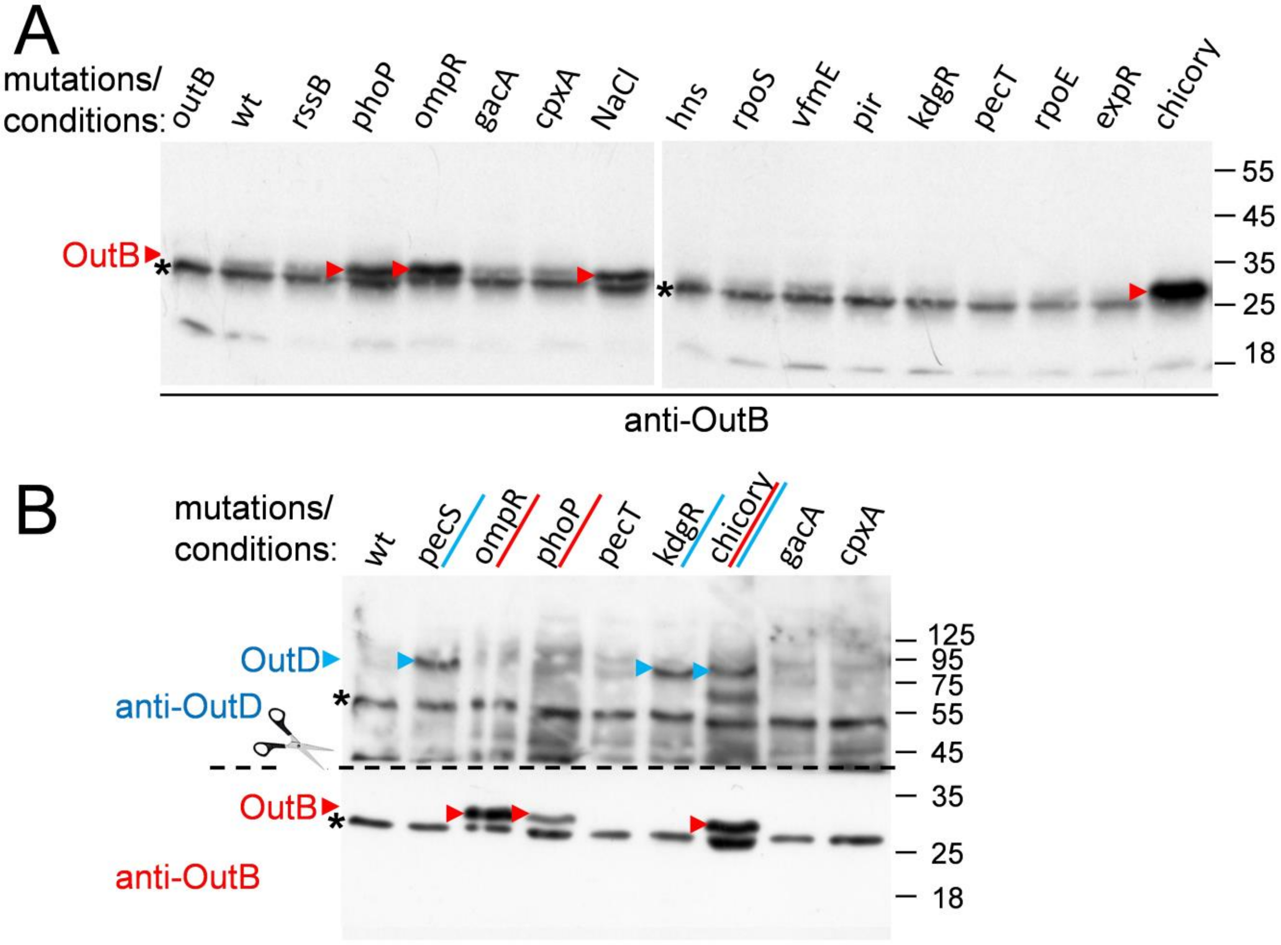

Figure S14. Expression of *outB* and *outD* is not co-regulated by the same regulatory genes. (**A**) OutB level is increased at high osmolarity, in the infected plant tissue and in the *ompR* and *phoP* regulatory mutants. Cell extracts from the indicated *D. dadantii* mutants were separated on SDS-PAGE and analyzed by Western with anti-OutB antibodies. Bacteria were grown in either LB (12 mM NaCl) or LB supplemented with 0.2 M NaCl, "NaCl" line. In "chicory", bacteria were grown in chicory leaves. An equivalent of 10^7 cells was loaded onto each line. OutB is shown with red arrows. Non-specific species are noted with an asterisk. (**B**) Comparison of OutB and OutD levels in *D. dadantii* regulatory mutants Cell extracts from the indicated *D. dadantii* mutants grown in LB supplemented with 0.2 % glycerol at 28°C for 14 h were separated on SDS-PAGE. An equivalent of 10^7 cells was loaded onto each line. After transfer, the upper and lower parts of the blot were probed separately, with anti-OutD or anti-OutB, respectively. The positions of OutD and OutB are shown with blue and red arrows, respectively. Non-specific species are noted with an asterisk. In "chicory", bacteria were grown in chicory leaves.

Figure S15. Original uncropped Western blots and gels supporting the main and supplementary figures.

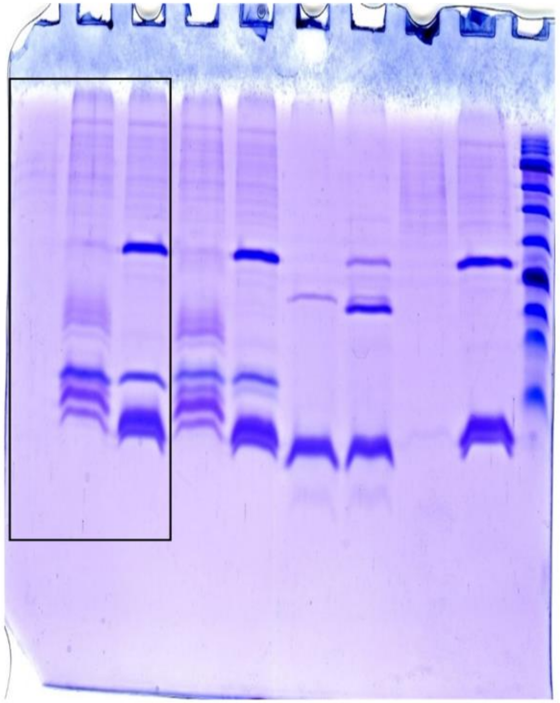

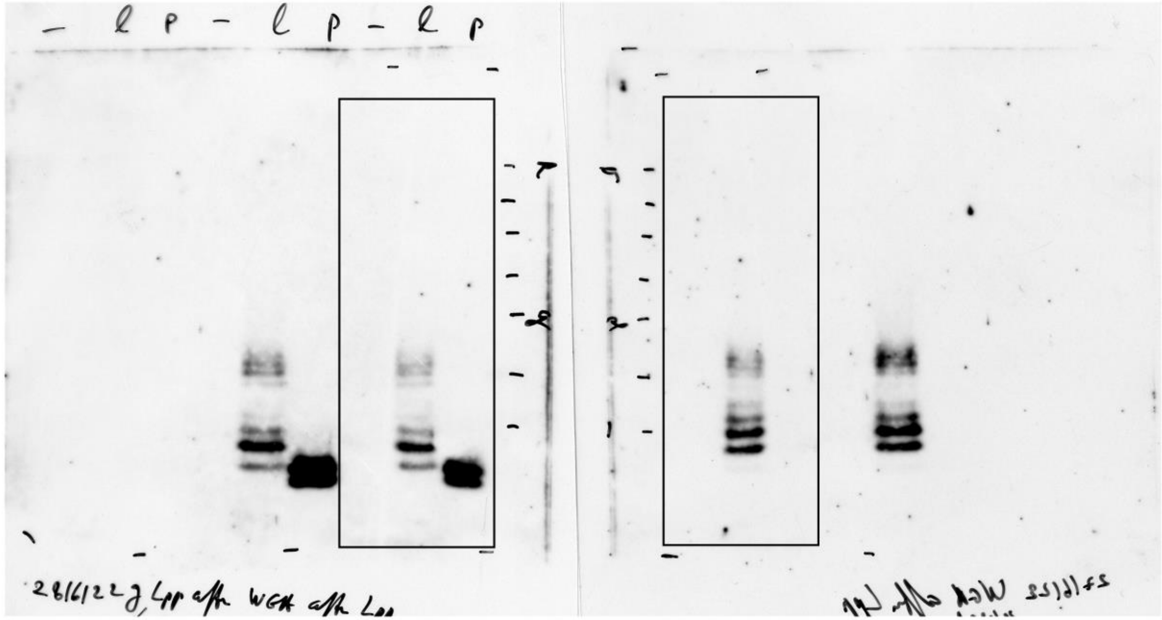

Figure 1B

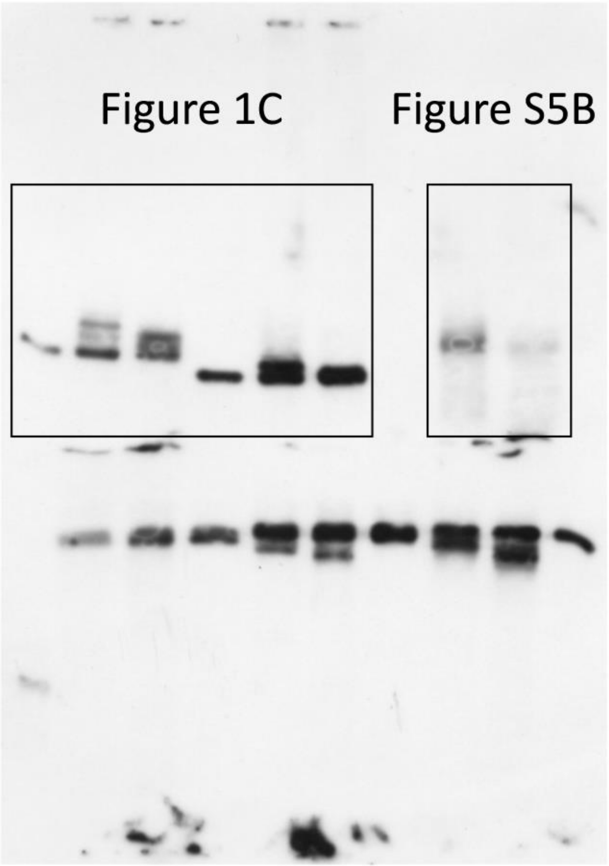
Figure 1C
Figure S5B

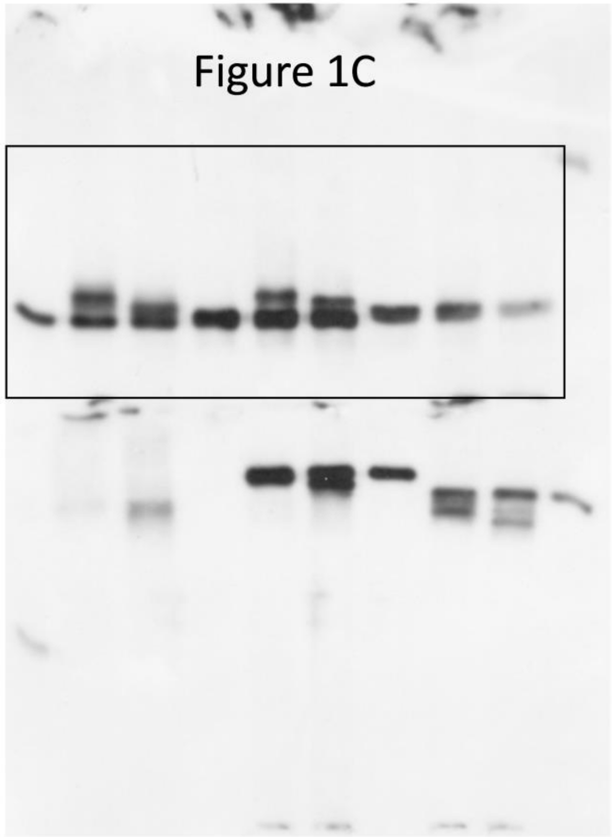
Figure 1C

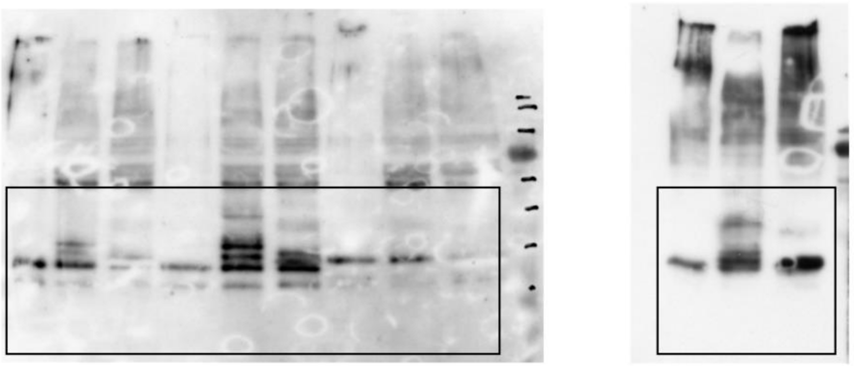

Figure 2B

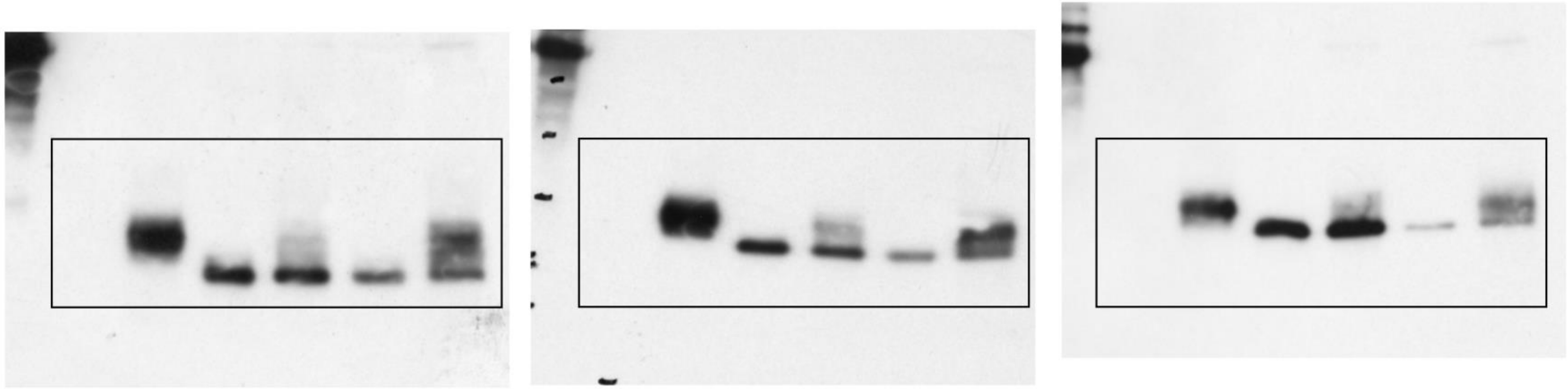

Figure 3C

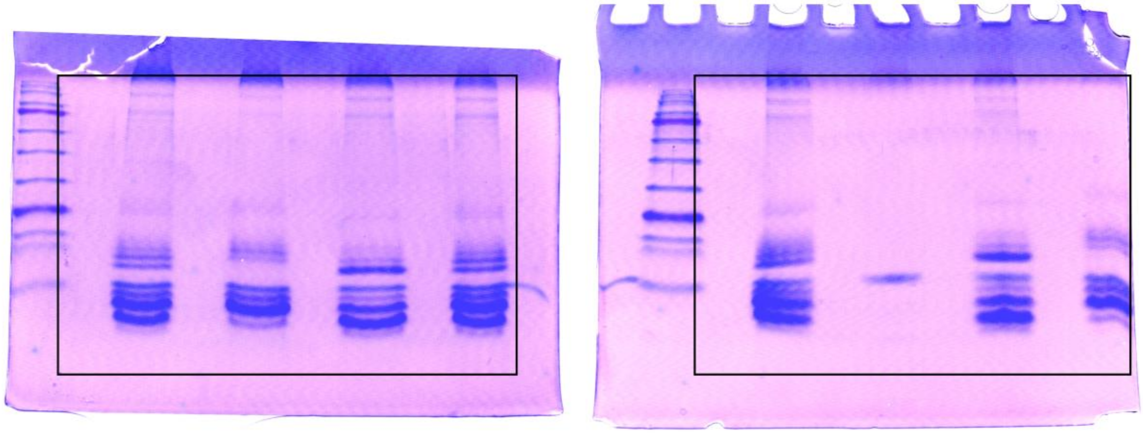

Figure 4A

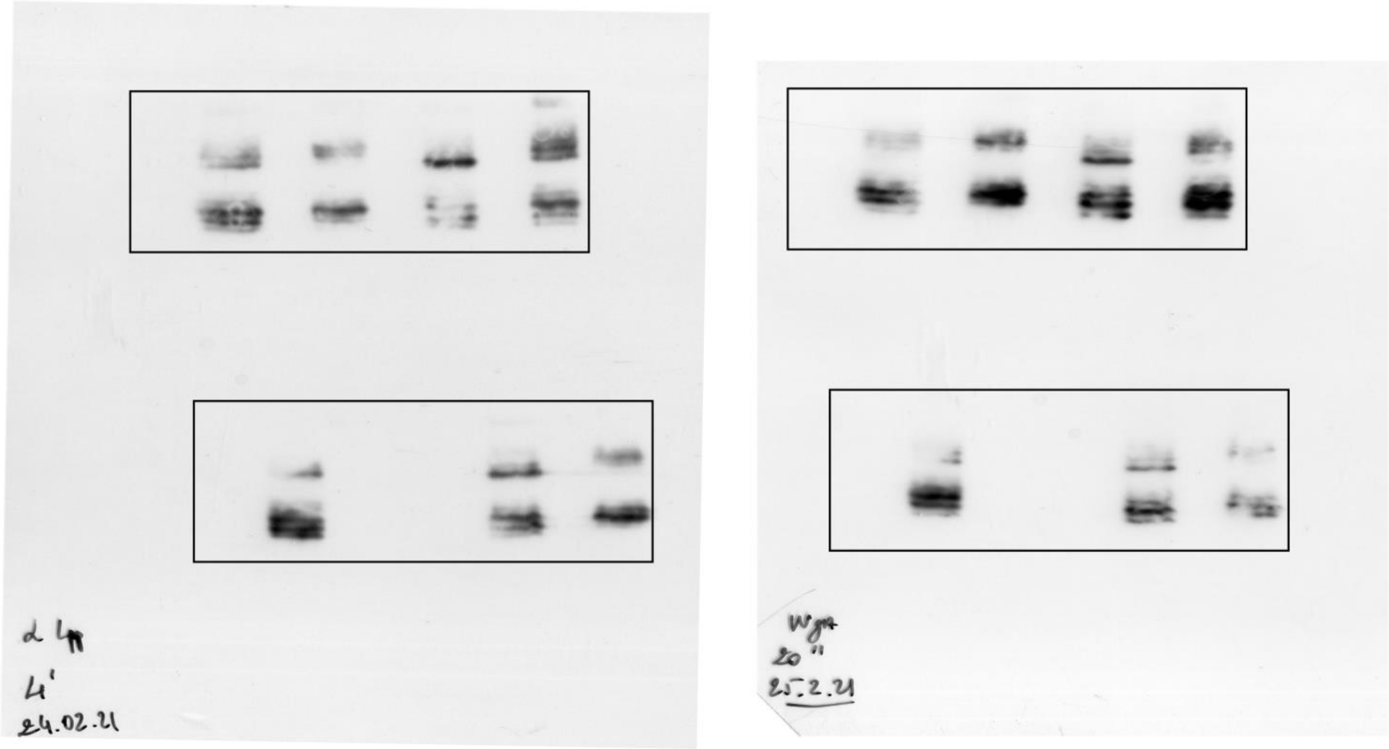

Figure 4A

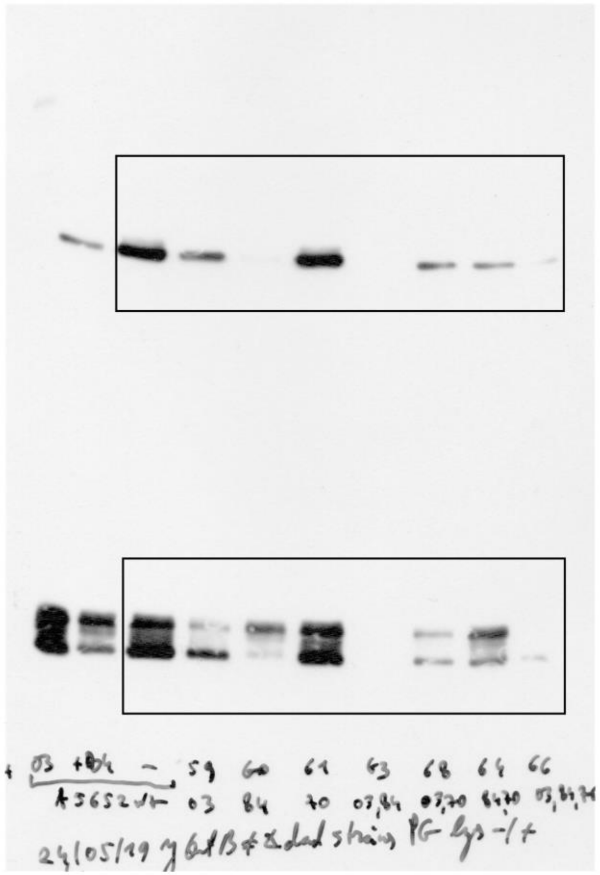

Figure 4B

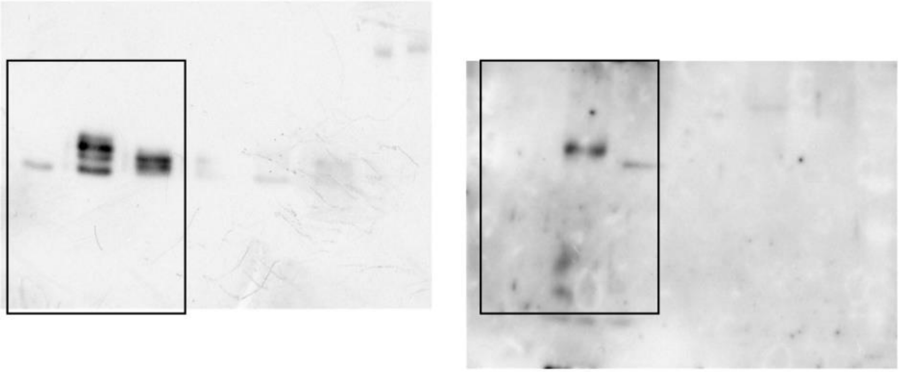

Figure S3

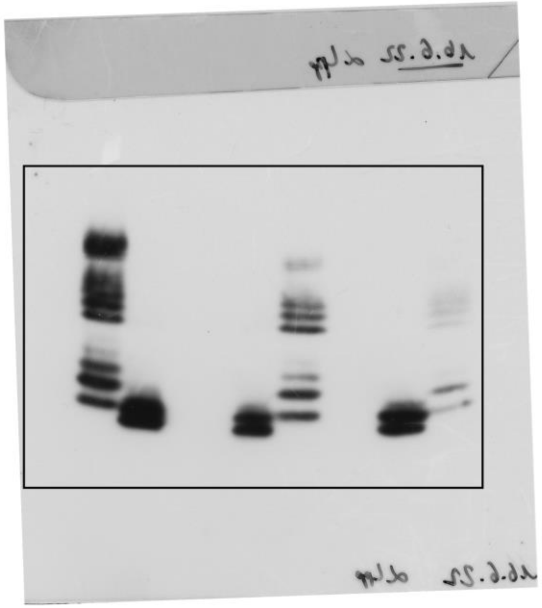

Figure S4B

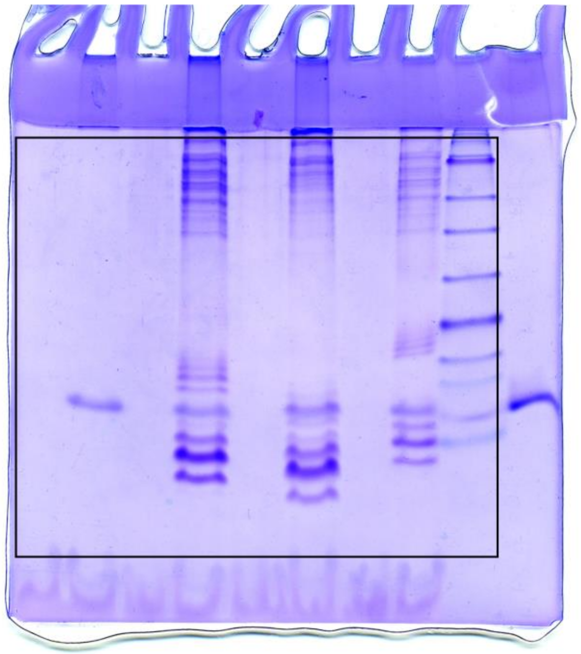

Figure S6C

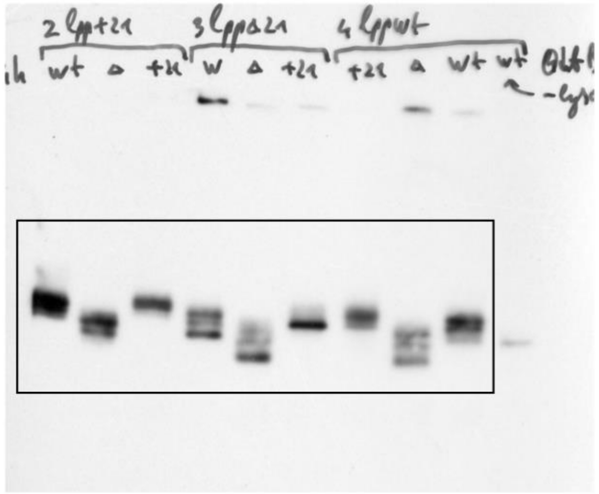

Figure S9B

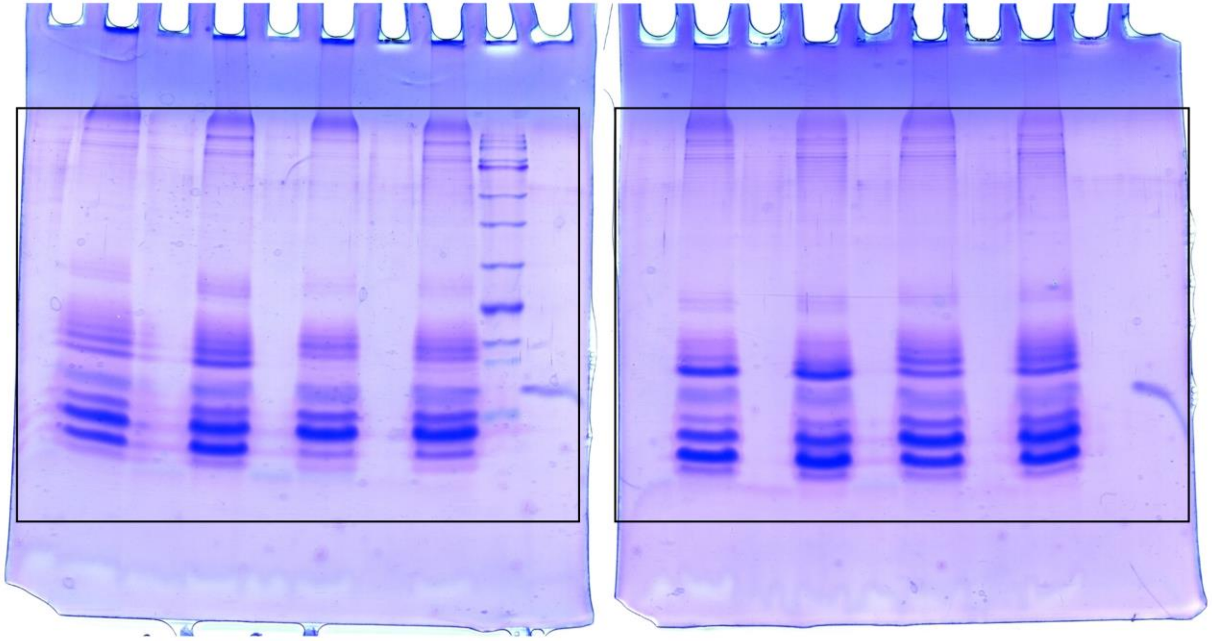

Figure S11A

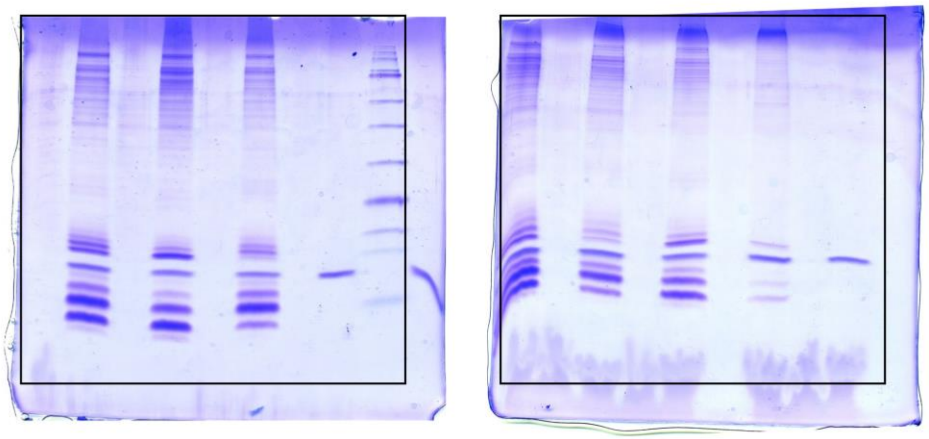

Figure S12A

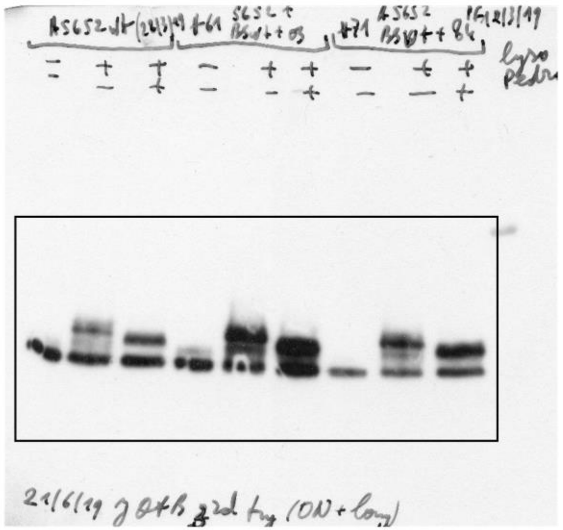

Figure S12B

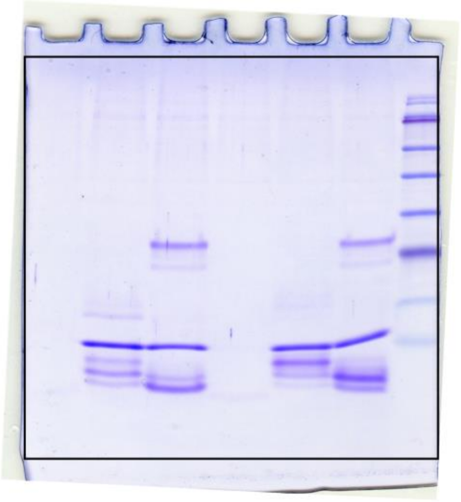

Figure S13C

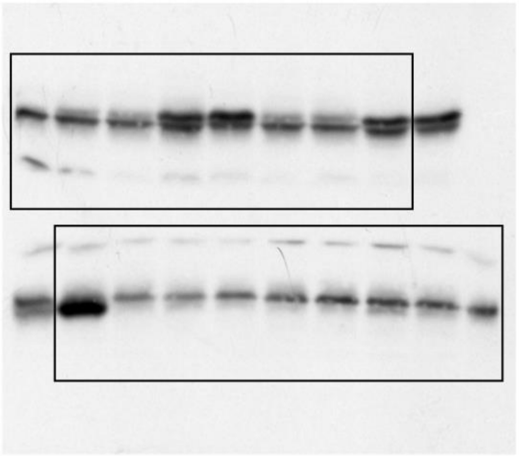

Figure S14A

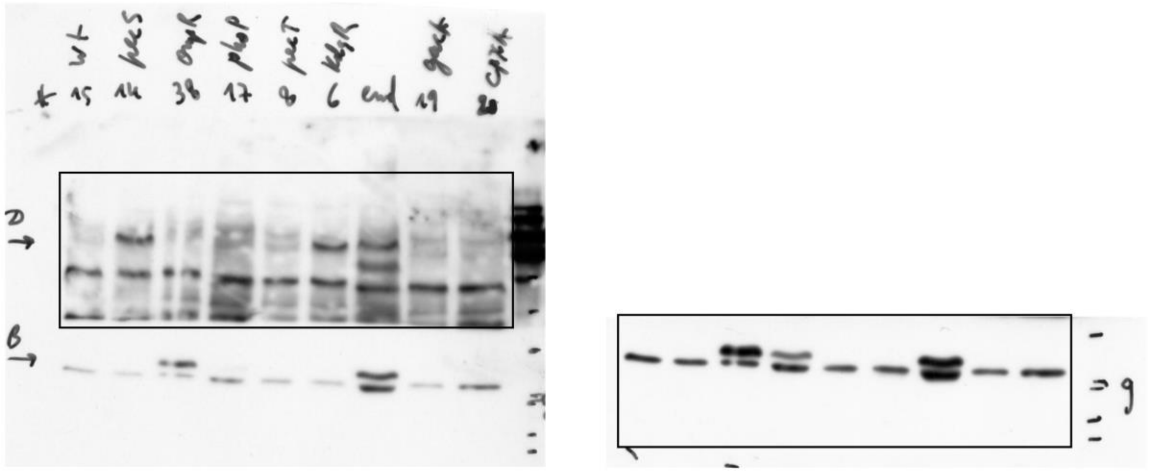

Figure S14B

Table S1

Muropeptide	Cross-link	Peak	Relative Abundance (%)	Monoisotopic mass	
				Calculated	Observed
GM^{Red}-Tri 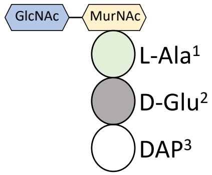	NA	1	13.5	870.371	870.383
GM^{Red}-Tri-Gly 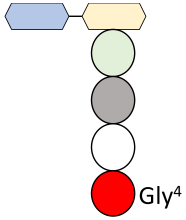	NA	2	1.6	927.392	927.393
GM^{Red}-Tetra-Gly 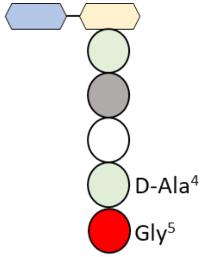	NA	3	1.3	998.429	998.429
GM^{Red}-Tetra 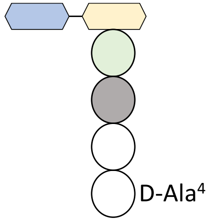	NA	4	37.2	941.408	941.404

GM^{Red}-Tri→Lys 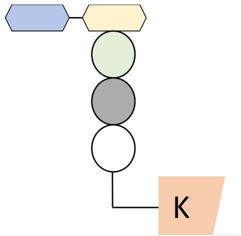	NA	5	2.4	998.466	998.461
GM^{Red}-Tri→Lys-Lys 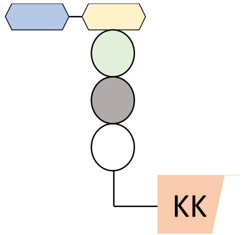	NA	6	1.6	1126.561	1126.561
GM-GM^{Red}-Tetra 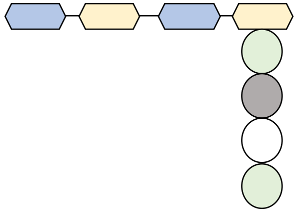	NA	7	2.5	1419.588	1419.588
GM^{Red}-Tri→GM^{Red}-Tri 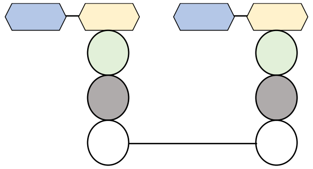	(3-3)	8	0.6	1722.731	1722.730

GM^{Red}-Tetra→GM^{Red}-Tri-Gly 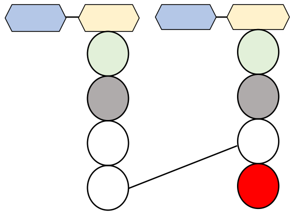	(4-3)	9	1.4	1850.789	1850.788
GM^{Red}-Tetra→GM^{Red}-Tri 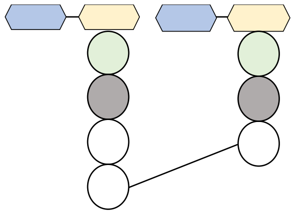	(4-3)	10	3.3	1793.768	1793.767
GM^{Red}-Tri→GM^{Red}-Tetra 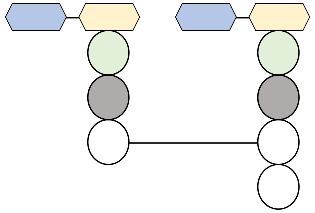	(3-3)	11	2.5	1793.768	1793.767
GM^{Red}-Tetra→GM^{Red}-Tri→Lys 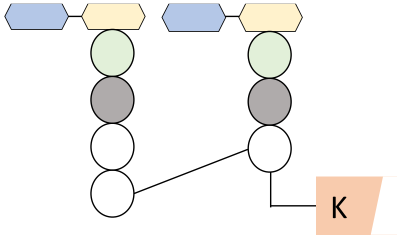	(4-3)	11	0.9	1921.863	1921.865

GM^{Red}-Tetra→GM^{Red}-Tetra 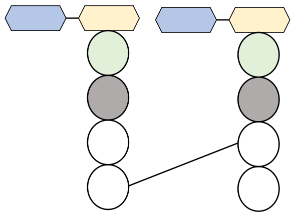	(4-3)	12	20.0	1864.805	1864.803
GM^{Red}-Tetra→GM^{Red}-Tri→Lys-Lys 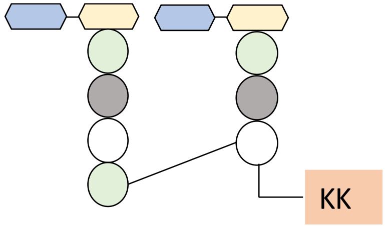	(4-3)	13	2.1	2049.958	2049.958
GM-GM^{Red}-Tetra→GM^{Red}-Tetra 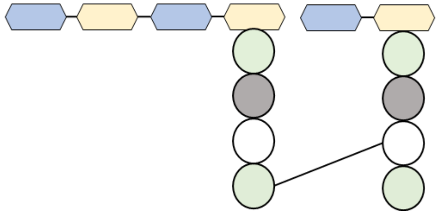	(4-3)	14	1.6	2342.986	2342.985
GM^{Anh}-Tetra→GM^{Red}-Tri<br>(One anhydro MurNAc residue) 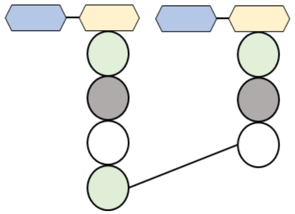	(4-3)	15	0.2	1773.741	1773,742

GM^{Anh}-Tetra→GM^{Red}-Tetra<br>(One MurNAc residue is not reduced)<br>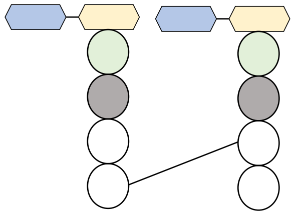	(4-3)	15	2.0	1862.788	1862.789
GM^{Anh}-Tetra<br>(One anhydro MurNAc residue)<br>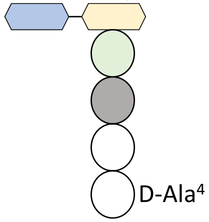	NA	16	3.0	921.383	921.381
GM^{Red}-Tetra→GM^{Anh}-Tetra<br>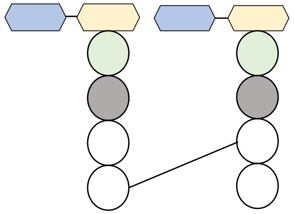	(4-3)	17	2.5	1844.779	1844.778

GM^{Red}-Tetra→GM^{Red}-Tetra→GM^{Red}-Tri→Lys-Lys 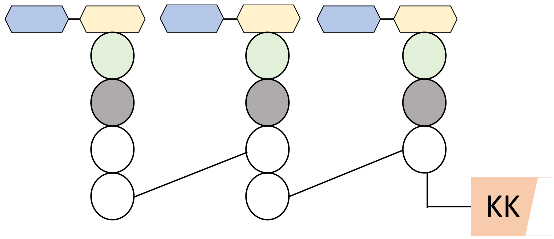	(4-3)<br>(4-3)	17	0.4	2973.355	2973.358
GM^{Red}-Tetra→GM^{Red}-Tetra→GM^{Red}-Tetra 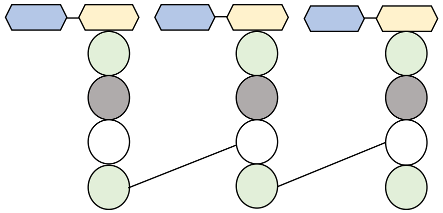	(4-3)<br>(4-3)	17	0.8	2788.205	2788.202

Table S1. Chemical structure of the main muropeptides from *Dickeya dadantii* 3937. The muropeptides were separated by the *rp*HPLC (shown on Supplementary Figure S1) and the relative abundance of molecular species found in the chromatographic peak were determined using their respective ion current intensities as measured by mass spectrometry. Abbreviations: Anh, anhydro; Red, reduced. The relative abundance of peptidoglycan fragments was estimated as the percentage of the total integrated area (λ = 205 nm).

Table S2. Bacterial strains and plasmids used in this study.

Strains/Plasmids	Genotype/phenotype	Source
Escherichia coli		
NM522	*supE thi-1* Δ(*lac-proAB*) Δ(*mcrB-hsdSM*)*5* (r_K^- m_K^+) [F´ *proAB lacI*^{q}*Z*Δ*M15*]	NEB
Dickeya dadantii 3937		
A5652	wild type	laboratory collection
A5654	*outB::uidA-nptI* (Km^R)	[1]
A6259	*ldt03::tetA* (Tc^R)	This work
A6260	*ldt84::aadA* (Sp^R/Sm^R)	This work
A6261	*ldt70::cat* (Cm^R)	This work
A6263	*ldt03::tetA ldt84::aadA* (Tc^R Sp^R/Sm^R)	This work
A6264	*ldt70::cat ldt84::aadA* (Cm^R Sp^R/Sm^R)	This work
A6266	*ldt03::tetA ldt84::aadA ldt70::cat* (Tc^R Sp^R/Sm^R Cm^R)	This work
A6267	*ldt03::tetA ldt70::cat* (Tc^R Cm^R)	This work
A6300	*ldt03::tetA ldt84::aadA ldt23::aacC* (Tc^R Sp^R/Sm^R Gm^R)	This work
A6301	*ldt23::aacC* (Gm^R)	This work
A6302	*ldt03::tetA ldt23::aacC* (Tc^R Gm^R)	This work
A6303	*ldt84::aadA ldt23::aacC* (Sp^R/Sm^R Gm^R)	This work
A6304	*ldt70::cat ldt23::aacC* (Gm^R Cm^R)	This work
A6589	*ldt03::tetA ldt84::aadA lpp::cat* (Tc^R Sp^R/Sm^R Cm^R)	This work
A6752	*lpp+21 cat* (Cm^R) inserted between *lpp+21* and *ldt84*	This work
A6753	*lpp*Δ*21 cat* (Cm^R) inserted between *lpp*Δ*21* and *ldt84*	This work
A6754	*lpp_wt cat* (Cm^R) inserted between *lpp* and *ldt84*	This work
A6761	*lpp::cat* (Cm^R)	This work
A3996	*rssB::cat* (Cm^R)	Sylvie Reverchon
A4109	*rpoS::cat* (Cm^R)	[2]
A4111	*vfmE::cat* (Cm^R)	[3]
A4112	*hns::nptI* (Km^R)	[4]
A4113	*pir::cat* (Cm^R)	Sylvie Reverchon
A4114	*kdgR::aadA* (Sp^R/Sm^R)	Sylvie Reverchon
A4116	*pecT::cat* (Cm^R) transduced from *D. dadantii* A2174[5]	laboratory collection
A4134	*rpoE::nptI* (Km^R)	Sylvie Reverchon
A4137	*phoP::cat* (Cm^R) transduced from *D. dadantii* A4194[6]	laboratory collection
A4165	*expR::uidA-nptI* (Km^R)	[7]
A4239	*gacA::cat* (Cm^R)	[8]
A4567	*ompR::tetA* (Tc^R)	[9]
A5627	*cpxA::aacC* (Gm^R) transduced from *D. dadantii* NFB7515[10]	laboratory collection
**Plasmids**		
pGEM-T	P*lac*, P*T7pol*, *blaM* (Ap^R), ColE1 origin	Promega
pBAD33	P_{BAD}, *cat* (Cm^R), p15A origin	[11]
pBS	Bluescript KS+, *blaM* (Ap^R), ColE1 origin	Stratagene

pBS-Km	Bluescript KS+, *neo* (Km^R), ColE1 origin	This work
pET-20b(+)	P*T7pol*, coding PelB signal peptide and 6His, *blaM* (Ap^R)	Novagen
BS	pGEM-T carrying *outB-outS* 1.6 kb fragment	This work
pGEM-T_Ldt03	pGEM-T carrying *ldt03* gene of *D. dadantii* under P*lacZ*	This work
pGEM-T_Ldt23	pGEM-T carrying *ldt23* gene of *D. dadantii* under P*lacZ*	This work
pGEM-T_Ldt70	pGEM-T carrying *ldt70* gene of *D. dadantii* under P*lacZ*	This work
pGEM-T_Ldt84	pGEM-T carrying *ldt84* gene of *D. dadantii* under P*lacZ*	This work
pBAD_Ldt03	pBAD33 carrying *ldt03* under P_{BAD}	This work
pBAD_Ldt84	pBAD33 carrying *ldt84* under P_{BAD}	This work
pBS_Ldt03	pBS-Km carrying *ldt03* under P*lacZ*	This work
pBS_Ldt84	pBS-Km carrying *ldt84* under P*lacZ*	This work
pGEM-T_Lpp	pGEM-T carrying the *lpp* gene of *D. dadantii* under P*lacZ*	This work
pBAD_Lpp	pBAD33 carrying the *lpp* gene of *D. dadantii* under P_{BAD}	This work
pGEM-T_LdtA	pGEM-T carrying the *ldtA (erfK)* gene of *E. coli* under P*lacZ*	This work
pGEM-T_LdtB	pGEM-T carrying the *ldtB (ybiS)* gene of *E. coli* under P*lacZ*	This work
pGEM-T_LdtC	pGEM-T carrying the *ldtC (ycfS)* gene of *E. coli* under P*lacZ*	This work

Table S3. Primers used in this study.

Primer	Nucleotide sequence (5’-3’) [a]	Generated mutation or cloned gene
OuB_T218Y[b]	cggagcaaaccgtcaggaca**tac**aagaaatgacacagcaactgc	OutB_T218Y
OuB_K219TGA[b]	gcaaaccgtcaggacaacg**tga**aaatgacacagcaactgcac	OutB_K219tga
OuB_K220tga	gaag**tg**atgacacagcaactgcacatc	OutB_K220tga
ROuB_K220tga	gtcat**ca**cttcgttgtcctgacggtttg	OutB_K220tga
OuB_Msc[b]	gattcgttgcaggattggcc**a**ggcgggaaaccgggcgatg	OutB_*Msc*I over P191-G192
Bla_Msc[b]	cctcactgattaagcattgg**cc**actgtcagaccaagtttactc	BlaM_*Msc*I over W286
BladltB	gacggtttgcggtttcccgcctggccaatg	OutB_ΔG196-E212=OutBΔC
RBladltB	gggaaaccgcaaaccgtcaggacaacgaag	OutB_ΔG196-E212=OutBΔC
OuBdltlink	ccgctgtcgactgcggatgaaaaccatcc	OutB_ΔS51-T72 = OutBΔ22
ROuBdltlink	catccgcagtcgacagcggaatggggctg	OutB_ΔS51-T72 = OutBΔ22
OuB_Sfo[b]	ccgcctaccaaagtgggc**gcc**gttccggcatcaacagcgg	OutB_*Sfo*I over G63-M64
For_6403	gtaccggataacaacaattcc	*ldt03*
Rev_6403	gcagcgtaagctgccctgtg	*ldt03*
For20084	gtaaccctgtgttgcgctctg	*ldt84*
Rev20084	gatgtcagaacgcccgctgatg	*ldt84*
For_20070	cgcgatgtgataaaagcagtg	*ldt70*
Rev_20070	gcccgtcgatgcaacccgag	*ldt70*
F46523	cctgcggcttgaaggatgtc	*ldt23*
RC_46523	ctcagccgttgaccggcaac	*ldt23*
F_LppRBS	caatttagagggtattaataatg	*lpp*
Rev_Lpp	tggcgcacaaagtgcgccat	*lpp*
Lpp_Xho[b]	ggttgctccagcaatgctaaact**c**ga**g**cagctgtcttctgacgtttcttc	Lpp_*Xho*I over L7-D8[c]
Lpp_SalI[b]	gctctgaccagcaaagt**c**gacgctctggctaccg	Lpp_*Sal*I over V28-D29[c]
LppK57R[b]	caaccaggttcgtacttaca**g**gaagtaagaactggttgaatg	Lpp_K57R[c]
LppY56T[b]	cctggacaaccaggttcgtact**ac**caagaagtaagaactggttg	Lpp_Y56T[c]
Lpp57	caag**t**agtaagaactggttgaatgaaaaatg	Lpp_Δ58K[c]
RLpp57	cttact**a**cttgtaagtacgaacctggttgtcc	Lpp_Δ58K[c]
Lpp_dlt58_Y56T	gtact**ac**caagtagtaagaactggttgaatg	Lpp_Y56T_Δ58K[c]
RLpp_dlt58_Y56T	cttg**gt**agtacgaacctggttgtccagg	Lpp_Y56T_Δ58K[c]
Lpp_strp	gcaat**tggagccacccccagttcgaaaaa**gctaaactggatcagctgtct	Lpp_Strep[d]
RLpp_strp	ttagctt**tttcgaactgggggtggctcca**attgctggagcaaccagccag	Lpp_Strep[d]
LdtA-for	gaattcgctatacttaacggatagc	*ldtA (erfK)*
LdtA-rev	aagctttctaattaccaacgc	*ldtA (erfK)*
LdtB-for	gaattccaatactctaatattctc	*ldtB (ybiS)*
LdtB-rev	aagcttatgagtccgccttttgctt	*ldtB (ybiS)*
LdtC-for	gaattcaggcttatctgtttattac	*ldtC (ycfS)*
LdtC-rev	aagcttccccgcgacatgccgtgtcg	*ldtC (ycfS)*

[a] Mutated or introduced bases are in bold.

[b] These primers were used together with corresponding reverse complementary primers (not shown).

[c] The Lpp residue numbering is this for the matured, signal peptide-less Lpp.

[d] Lpp_Strep carries the WSHPQFEK sequence between Asn4 & Ala5 of the matured Lpp.

Supplementary Methods: Plasmid and strain construction.

To generate OutB+21, a *Sfo*I site was introduced into the *outB* sequence covering the codons of G63 and M64. Next, the *Eco*RI-*Sfo*I *outB* fragment encoding the residues M1 to G63 was fused to the *Eco*RV-*Eco*RI *outB* fragment encoding the residues I43 to K220. In this way, in OutB+21, the sequence I43 to G63 was repeated twice. OutBΔ22 variant missing residues S51 to T72 was generated by PCR using OuBdltlink and ROuBdltlink primers. OutBΔC variant missing residues G196-E212 was generated by PCR using BladltB and RBladltB primers. To generate BlaM-CTE fusion in pGEM-T vector, an *Msc*I site was introduced into the *blaM* sequence covering the W286 codon. Next the *Ssp*I-*Msc*I *blaM* fragment from pGEM-T was inserted into *Sma*I-*Msc*I sites of the BS plasmid in the place of *outB* sequence encoding residues M1 to W190. In this way, full-length BlaM was fused to P191-K220 fragment of OutB.

To generate LppΔ21, an *Xho*I and a *Sal*I sites were introduced into the *lpp* sequence, covering respectively, the codons of L7-D8 and V28-D29 of the mature, signal peptide-less Lpp. Next, the *Xho*I-*Sal*I *lpp* fragment encoding residues D8 to V28 was deleted, producing *lppΔ21*. To generate Lpp+21, the *Xho*I-*Sac*II *lpp* fragment encoding residues Q9 to K58 was fused to the *Sal*I-*Sac*II *lpp* fragment encoding residues C1 to E29. In this way, in Lpp+21, the sequence Q9 to E29 was repeated twice.

Mutant *D. dadantii* strains carrying chromosomal mutations in *ldt*, *lpp* or *outB* genes were generated by by homologous recombination. To this end, a *tetA* (Tc^R) cartridge from pHP45Ω-Tc plasmid was introduced into the unique *Eco*RI site of *ldt03*, an *aadA* (Sp^R/Sm^R) cartridge from pHP45Ω-Sm plasmid was introduced into the unique *Bam*HI site of *ldt84*; an *aacC* (Gm^R) cartridge from p34S-Gm plasmid was introduced into the unique *Bam*HI site of *ldt23*, and a *cat* (Cm^R) cartridge from pCKC15 plasmid was introduced into the unique *Age*I site of *ldt70* and into the unique *Hinc*II site of *lpp* [12–14].

Construction of *lpp*, *lppΔ21* and *lpp+21* mutant strains is shown in Fig. S6. The *cat* (Cm^R) gene, inserted between the *lpp* and *ldt84* genes was used as a selective marker for *de novo* transductions of the mutated *lpp* alleles into the *D. dadantii* wild type.

Supplementary references

1. Zhang, S. *et al.* Scaffolding Protein GspB/OutB Facilitates Assembly of the Dickeya dadantii Type 2 Secretion System by Anchoring the Outer Membrane Secretin Pore to the Inner Membrane and to the Peptidoglycan Cell Wall. *mBio* **13**, e0025322 (2022).

2. Boughammoura, A. *et al.* Differential role of ferritins in iron metabolism and virulence of the plant-pathogenic bacterium Erwinia chrysanthemi 3937. *J. Bacteriol.* **190**, 1518–1530 (2008).

3. Nasser, W. *et al.* Vfm a new quorum sensing system controls the virulence of Dickeya dadantii. *Environ. Microbiol.* **15**, 865–880 (2013).

4. Nasser, W., Faelen, M., Hugouvieux-Cotte-Pattat, N. & Reverchon, S. Role of the nucleoid-associated protein H-NS in the synthesis of virulence factors in the phytopathogenic bacterium Erwinia chrysanthemi. *Mol. Plant-Microbe Interact. MPMI* **14**, 10–20 (2001).

5. Surgey, N., Robert-Baudouy, J. & Condemine, G. The Erwinia chrysanthemi pecT gene regulates pectinase gene expression. *J. Bacteriol.* **178**, 1593–1599 (1996).

6. Costechareyre, D., Chich, J.-F., Strub, J.-M., Rahbé, Y. & Condemine, G. Transcriptome of Dickeya dadantii infecting Acyrthosiphon pisum reveals a strong defense against antimicrobial peptides. *PloS One* **8**, e54118 (2013).

7. Nasser, W., Bouillant, M. L., Salmond, G. & Reverchon, S. Characterization of the Erwinia chrysanthemi expI-expR locus directing the synthesis of two N-acyl-homoserine lactone signal molecules. *Mol. Microbiol.* **29**, 1391–1405 (1998).

8. Lebeau, A. *et al.* The GacA global regulator is required for the appropriate expression of Erwinia chrysanthemi 3937 pathogenicity genes during plant infection. *Environ. Microbiol.* **10**, 545–559 (2008).

9. Condemine, G. & Ghazi, A. Differential regulation of two oligogalacturonate outer membrane channels, KdgN and KdgM, of Dickeya dadantii (Erwinia chrysanthemi). *J. Bacteriol.* **189**, 5955–5962 (2007).

10. Bontemps-Gallo, S., Madec, E. & Lacroix, J.-M. The two-component system CpxAR is essential for virulence in the phytopathogen bacteria Dickeya dadantii EC3937. *Environ. Microbiol.* **17**, 4415–4428 (2015).

11. Guzman, L. M., Belin, D., Carson, M. J. & Beckwith, J. Tight regulation, modulation, and high-level expression by vectors containing the arabinose PBAD promoter. *J. Bacteriol.* **177**, 4121–4130 (1995).

12. Dennis, J. J. & Zylstra, G. J. Plasposons: modular self-cloning minitransposon derivatives for rapid genetic analysis of gram-negative bacterial genomes. *Appl. Environ. Microbiol.* **64**, 2710–2715 (1998).

13. Fellay, R., Frey, J. & Krisch, H. Interposon mutagenesis of soil and water bacteria: a family of DNA fragments designed for in vitro insertional mutagenesis of gram-negative bacteria. *Gene* **52**, 147–154 (1987).

14. Prentki, P. & Krisch, H. M. In vitro insertional mutagenesis with a selectable DNA fragment. *Gene* **29**, 303–313 (1984).
