# Supplementary material for: An inner membrane protein is covalently attached to peptidoglycan in the γ-proteobacterium Dickeya dadantii
Source: Commun Biol. 2025 Jul 18;8:1071. doi: 10.1038/s42003-025-08488-9 (PMC12274547; doi:10.1038/s42003-025-08488-9)
Supplement: Supplementary file 4 — Reporting summary [file 42003_2025_8488_MOESM4_ESM.pdf]

nature portfolio

Corresponding author(s): Vladimir E. Shevchik

Last updated by author(s): Jun 24, 2025

Reporting Summary

Nature Portfolio wishes to improve the reproducibility of the work that we publish. This form provides structure for consistency and transparency in reporting. For further information on Nature Portfolio policies, see our Editorial Policies and the Editorial Policy Checklist.

Statistics

For all statistical analyses, confirm that the following items are present in the figure legend, table legend, main text, or Methods section.

n/a	Confirmed	
☐	☒	The exact sample size (*n*) for each experimental group/condition, given as a discrete number and unit of measurement
☐	☒	A statement on whether measurements were taken from distinct samples or whether the same sample was measured repeatedly
☐	☒	The statistical test(s) used AND whether they are one- or two-sided<br>*Only common tests should be described solely by name; describe more complex techniques in the Methods section.*
☒	☐	A description of all covariates tested
☒	☐	A description of any assumptions or corrections, such as tests of normality and adjustment for multiple comparisons
☐	☒	A full description of the statistical parameters including central tendency (e.g. means) or other basic estimates (e.g. regression coefficient) AND variation (e.g. standard deviation) or associated estimates of uncertainty (e.g. confidence intervals)
☐	☒	For null hypothesis testing, the test statistic (e.g. *F*, *t*, *r*) with confidence intervals, effect sizes, degrees of freedom and *P* value noted<br>*Give P values as exact values whenever suitable.*
☒	☐	For Bayesian analysis, information on the choice of priors and Markov chain Monte Carlo settings
☒	☐	For hierarchical and complex designs, identification of the appropriate level for tests and full reporting of outcomes
☒	☐	Estimates of effect sizes (e.g. Cohen's *d*, Pearson's *r*), indicating how they were calculated

Our web collection on statistics for biologists contains articles on many of the points above.

Software and code

Policy information about availability of computer code

Data collection	Evolution Capt Edge (Vilber Lourmat), for immunoblotting; Radius EM Imaging Software (Emsis), for TEM
Data analysis	PRISM 10.0 (GraphPadSoftware); ImageJ 1,53k (NIH); Evolution Capt Edge (Vilber Lourmat), ProteoWizard (10.1038/nbt.2377); mineXpert2 (10.1021/jasms.0c00402)

For manuscripts utilizing custom algorithms or software that are central to the research but not yet described in published literature, software must be made available to editors and reviewers. We strongly encourage code deposition in a community repository (e.g. GitHub). See the Nature Portfolio guidelines for submitting code & software for further information.

Data

Policy information about availability of data

All manuscripts must include a data availability statement. This statement should provide the following information, where applicable:

- Accession codes, unique identifiers, or web links for publicly available datasets
- A description of any restrictions on data availability
- For clinical datasets or third party data, please ensure that the statement adheres to our policy

All data of this study are presented in the main text, supplementary information and source data files. All bacterial strains are available upon request. The raw data of MS analyses have been deposited in Zenodo open repository under accession code https://zenodo.org/records/15691255.

Research involving human participants, their data, or biological material

Policy information about studies with human participants or human data. See also policy information about sex, gender (identity/presentation), and sexual orientation and race, ethnicity and racism.

Reporting on sex and gender	N/A
Reporting on race, ethnicity, or other socially relevant groupings	N/A
Population characteristics	N/A
Recruitment	N/A
Ethics oversight	N/A

Note that full information on the approval of the study protocol must also be provided in the manuscript.

Field-specific reporting

Please select the one below that is the best fit for your research. If you are not sure, read the appropriate sections before making your selection.

☒ Life sciences ☐ Behavioural & social sciences ☐ Ecological, evolutionary & environmental sciences

For a reference copy of the document with all sections, see nature.com/documents/nr-reporting-summary-flat.pdf

Life sciences study design

All studies must disclose on these points even when the disclosure is negative.

Sample size	For cell length measurements, 100 cell micrographs were randomly selected for each strain. All the collected values, including outliers, were taken for analysis. For immunoblotting analyses, at least five technical replicates of two biological experiments were performed.
Data exclusions	No data were excluded from the analyses.
Replication	Routinely, at least three independent biological replicas were analysed. The Western blots shown are representative of five technical replicates of at least two biological experiments.
Randomization	In the microscopy analyses, the images and the fields of analysed cells were selected randomly. No specific randomizations were necessary in all other experiments.
Blinding	When relevant, e.g., microscopy analyses, the data were blindly collected and analysed. In all other experiments, data analysis were not performed blindly to facilitate data storage and analysis.

Reporting for specific materials, systems and methods

We require information from authors about some types of materials, experimental systems and methods used in many studies. Here, indicate whether each material, system or method listed is relevant to your study. If you are not sure if a list item applies to your research, read the appropriate section before selecting a response.

Materials & experimental systems

n/a	Involved in the study	
☐	☒	Antibodies
☒	☐	Eukaryotic cell lines
☒	☐	Palaeontology and archaeology
☒	☐	Animals and other organisms
☒	☐	Clinical data
☒	☐	Dual use research of concern
☒	☐	Plants

Methods

n/a	Involved in the study	
☒	☐	ChIP-seq
☒	☐	Flow cytometry
☒	☐	MRI-based neuroimaging

Antibodies

Antibodies used	anti-rabbit goat IgG conjugated to peroxidase (A9169,Sigma-Aldrich); anti-BlaM (5 Prime-3 Prime, Inc); anti-OutB (described in 10.1128/mbio.00253-22), and anti-Lpp rabbit antibodies were genereted in this study using recombinant Lpp from Dickeya dadantii.
Validation	The specificity of anti-OutB and anti-Lpp antibodies was validated by immunoblotting with reference strains, either producing or not these proteins.

Plants

Seed stocks	N/A
Novel plant genotypes	N/A
Authentication	N/A
